# Outcomes based on prior therapy in the phase 3 METEOR trial of cabozantinib versus everolimus in advanced renal cell carcinoma

**DOI:** 10.1038/s41416-018-0164-0

**Published:** 2018-09-10

**Authors:** Thomas Powles, Robert J. Motzer, Bernard Escudier, Sumanta Pal, Christian Kollmannsberger, Joanna Pikiel, Howard Gurney, Sun Young Rha, Se Hoon Park, Poul F. Geertsen, Marine Gross-Goupil, Enrique Grande, Cristina Suarez, David W. Markby, Alan Arroyo, Mark Dean, Toni K. Choueiri, Daniel George

**Affiliations:** 1 Barts Cancer Institute, Cancer Research UK Experimental Cancer Medicine Centre, Queen Mary University of London, Royal Free NHS Trust, Queen Mary, University of London, London, E1 4NS UK; 2 Memorial Sloan Kettering Cancer Center, New York, NY 10065 USA; 3 Gustave Roussy, 94 805 Villejuif Cedex, France; 4 City of Hope National Medical Center, Duarte, CA 91010 USA; 5 British Columbia Cancer Agency, BCCA Vancouver Cancer Centre, Vancouver, BC V5Z 4E6 Canada; 6 Wojewodzkie Centrum Onkologii, 80-219 Gdańsk, Poland; 7 Macquarie University and Westmead Hospital, Macquarie University, Sydney, NSW 2109 Australia; 8 Severance Hospital, Yonsei Cancer Center, Seodaemun-Gu, Seoul, 03722 South Korea; 9 Samsung Medical Center, Seoul, South Korea; 10 Herlev and Gentofte Hospital, Herlev Hospital, Copenhagen University, Herlev, Denmark; 11 Groupe Hospitalier Saint André, 33000 Bordeaux, France; 12 Hospital Universitario Ramón y Cajal, Ctra. Colmenar Viejo, km. 9,100, 28034 Madrid, Spain; 13 Vall d’Hebron University Hospital and Institute of Oncology, Universitat Autònoma de Barcelona, Passeig de la Vall d’Hebron, 119-129, 08035 Barcelona, Spain; 14 Exelixis, Inc, South San Francisco, CA 94080 USA; 15 Dana-Farber Cancer Institute, Boston, MA 02215 USA; 16 Duke University Medical Center, Durham, NC 27710 USA

## Abstract

**Background:**

In the phase 3 METEOR trial, cabozantinib improved progression-free survival (PFS), objective response rate (ORR), and overall survival (OS) versus everolimus in patients with advanced renal cell carcinoma (RCC), after prior antiangiogenic therapy.

**Methods:**

Outcomes were evaluated for subgroups defined by prior therapy with sunitinib or pazopanib as the only prior VEGFR inhibitor, or prior anti-PD-1/PD-L1 therapy.

**Results:**

For the prior sunitinib subgroup (*N* = 267), median PFS for cabozantinib versus everolimus was 9.1 versus 3.7 months (HR 0.43, 95% CI 0.32–0.59), ORR was 16% versus 3%, and median OS was 21.4 versus 16.5 months (HR 0.66, 95% CI 0.47–0.93). For the prior pazopanib subgroup (*N* = 171), median PFS for cabozantinib versus everolimus was 7.4 versus 5.1 months (HR 0.67, 95% CI 0.45–0.99), ORR was 19% versus 4%, and median OS was 22.0 versus 17.5 months (HR 0.66, 95% CI 0.42–1.04). For prior anti-PD-1/PD-L1 therapy (*N* = 32), median PFS was not reached for cabozantinib versus 4.1 months for everolimus (HR 0.22, 95% CI 0.07–0.65), ORR was 22% versus 0%, and median OS was not reached versus 16.3 months (HR 0.56, 95% CI 0.21–1.52).

**Conclusions:**

Cabozantinib was associated with improved clinical outcomes versus everolimus in patients with advanced RCC, irrespective of prior therapy, including checkpoint inhibitor therapy.

## Introduction

During the past decade, treatments for advanced renal cell carcinoma (RCC) have evolved dramatically with the approval of multiple targeted therapies, including VEGF receptor tyrosine kinase inhibitors (VEGFR TKIs; sorafenib, sunitinib, pazopanib, axitinib), the VEGF-targeted monoclonal antibody bevacizumab, and mTOR inhibitors (temsirolimus and everolimus).^1,2^ Although survival of patients with advanced RCC has improved since the introduction of targeted therapy, current treatments are rarely curative and often result in therapeutic resistance.^1^ Long-term disease management has relied on sequential treatment with VEGF-targeted agents and mTOR inhibitors, with sunitinib and pazopanib as the most commonly used first-line therapies.^1,3,4^ Recent studies of nivolumab,^5^ cabozantinib,^6,7^ and lenvatinib in combination with everolimus^8^ supported approval of these regimens for previously treated patients; both nivolumab and cabozantinib demonstrated improved survival versus everolimus. Given the availability of many treatment options, it is important to consider the effect of prior therapy on patient outcomes to maximise clinical benefit in the second-line setting and beyond.

Cabozantinib, an orally bioavailable TKI, inhibits targets implicated in the pathogenesis and progression of RCC, including VEGFRs, MET, and AXL.^9,10^ Resistance to VEGF pathway inhibition is associated with activation of MET and AXL signalling, and cabozantinib overcomes sunitinib resistance in preclinical RCC models.^9^ The pivotal phase 3 METEOR trial evaluated cabozantinib versus everolimus in patients with advanced RCC following VEGFR TKI therapy.^6,7^ Compared with everolimus, cabozantinib demonstrated improved progression-free survival (PFS), overall survival (OS), and objective response rate (ORR). Here we describe subgroup analyses of clinical outcomes in the METEOR trial based on prior therapy with VEGFR TKIs and PD-1/PD-L1 immune checkpoint inhibitors.

## Methods

### Study design and patients

The study design and methods for the international, randomised, open-label, phase 3 METEOR trial (NCT01865747) have been previously reported.^6,7^ Key eligibility criteria included a diagnosis of RCC with a clear-cell component, measurable disease per response evaluation criteria in solid tumours (RECIST) v1.1,^11^ and prior treatment with ≥1 VEGFR TKI. Radiographic progression during or within 6 months of the most recent VEGFR TKI regimen was required. The last dose of VEGFR TKI must have been received between 6 months and 2 weeks before randomisation. Previous treatment with immune checkpoint inhibitors was permitted, and treatment must have been stopped >4 weeks before randomisation. Other allowed prior therapies included interferon-α, interleukin-2, bevacizumab, cytotoxic chemotherapy, nephrectomy, and radiotherapy; there was no limit to the number of prior therapies. Prior therapy with cabozantinib or an mTOR inhibitor was not permitted. Karnofsky Performance Status ≥ 70% and adequate organ function were required.

Six hundred and fifty-eight patients were randomised 1:1 to receive cabozantinib (60 mg once daily) or everolimus (10 mg once daily). Stratification was by Memorial Sloan Kettering Cancer Center (MSKCC) risk group^12^ (favourable, intermediate, or poor) and number of prior VEGFR TKIs (1 or ≥2). Dose reductions (to 40 mg and 20 mg for cabozantinib or to 5 mg and 2.5 mg for everolimus) were allowed to manage adverse events (AEs).

The study was conducted in accordance with the Good Clinical Practice guidelines and the Declaration of Helsinki. The protocol was approved by the ethics committee or institutional review board at each centre, and all patients provided written informed consent.

### Assessments

Measures of clinical outcome included the primary endpoint of PFS, the secondary endpoints of ORR and OS, and safety. Tumour assessment by computed tomography or magnetic resonance imaging was performed at screening, every 8 weeks for the first 12 months, and every 12 weeks thereafter. Tumour response was assessed by RECIST v1.1.^11^ PFS was per independent radiology committee (IRC). Safety was evaluated every 2 weeks for the first 8 weeks and every 4 weeks thereafter until treatment discontinuation. A follow-up visit was scheduled 30 days after treatment discontinuation. AEs were reported according to National Cancer Institute Common Terminology Criteria for Adverse Events, v4.0.^13^ Data cut-off dates were 22 May 2015 for PFS and ORR and 31 December 2015 for OS and safety.

### Statistical analysis

Efficacy and safety outcomes for METEOR, including hazard ratios (HRs) for PFS and OS for subgroups based on prior therapy, have been previously reported.^6,7^ Efficacy analyses used all randomised patients, and safety analyses used all patients who received ≥1 dose of study drug. PFS and OS were assessed by the Kaplan–Meier method. No adjustments for multiplicity were made for subgroup analyses. Confidence intervals are considered descriptive, and HRs are unstratified.

PFS, ORR, and OS were evaluated in subgroups defined by number of prior VEGFR TKI therapies (1 or ≥2) and duration of treatment with first VEGFR TKI (≤6 or >6 months). PFS, ORR, OS, and safety were evaluated in subgroups defined by treatment with sunitinib or pazopanib as the only prior VEGFR TKI and by treatment with prior anti-PD-1/PD-L1. Subgroup analyses of efficacy were prespecified except those based on prior treatment with sunitinib or pazopanib.

## Results

### Patients

From 8 August 2013 to 24 November 2014, 658 patients were randomised 1:1 to receive cabozantinib (*N* = 330) or everolimus (*N* = 328). Two hundred thirty-five (71%) patients in the cabozantinib arm and 229 (70%) patients in the everolimus arm had received only one prior VEGFR TKI. One hundred thirty-five (41%) patients in the cabozantinib arm and 132 (40%) in the everolimus arm had received sunitinib as their only prior VEGFR TKI, and 88 (27%) in the cabozantinib arm and 83 (25%) in the everolimus arm had received prior pazopanib only.

Thirty-two (4.9%) patients had received prior anti-PD-1/PD-L1 therapy, 18 (5.4%) in the cabozantinib arm and 14 (4.3%) in the everolimus arm (Supplement Figure S1). Thirty-one patients had received nivolumab and one had received atezolizumab **(**Supplement Table S1**)**. Most had received anti-PD-1/PD-L1 therapy in the second-line setting or later: 16 patients in the cabozantinib arm and 12 in the everolimus arm. Six patients in the cabozantinib arm and seven in the everolimus arm had received nivolumab as their most recent prior therapy. The median duration of prior therapy with PD-1/PD-L1 inhibitors was 18.0 weeks (range 4.4–62.6 weeks, *N* = 31; duration missing for one patient). Forty-nine (7.4%) patients had received prior IL-2 therapy, 20 (6.1%) in the cabozantinib arm and 29 (8.8%) in the everolimus arm.

Demographics and baseline characteristics were generally balanced between treatment groups for patients who had received sunitinib or pazopanib as the only prior VEGFR TKI **(**Table 1**)**, although for the prior pazopanib subgroup, patients in the cabozantinib group had better ECOG performance status compared with the everolimus group (75% versus 59% had ECOG PS 0). In the prior anti-PD-1/PD-L1 subgroup, patients in the cabozantinib group had less favourable MSKCC risk status compared with the everolimus group (28% versus 43% had favourable risk and 22% versus 7% had poor risk for cabozantinib versus everolimus) and had received ≥2 VEGFR TKI therapies more frequently (61% versus 43%, respectively).

**Table 1:** Baseline characteristics

	Prior sunitinib only		Prior pazopanib only		Prior anti–PD-1/PD-L1	
	Cabozantinib *N* = 135	Everolimus *N* = 132	Cabozantinib *N* = 88	Everolimus *N* = 83	Cabozantinib *N* = 18	Everolimus *N* = 14
Median age, years (range)	62.0 (37–79)	62.0 (31–81)	63.0 (38–86)	61.0 (36–84)	63.5 (47–81)	61.0 (37–84)
Sex, *n* (%)						
Male	106 (79)	95 (72)	68 (77)	64 (77)	13 (72)	11 (79)
Female	29 (21)	36 (27)	20 (23)	19 (23)	5 (28)	3 (21)
Missing	0	1 (1)	0	0	0	0
Enrollment region, *n* (%)						
Europe	72 (53)	66 (50)	34 (39)	34 (41)	6 (33)	6 (43)
North America	45 (33)	43 (33)	35 (40)	32 (39)	11 (61)	7 (50)
Asia Pacific	18 (13)	22 (17)	13 (15)	13 (16)	1 (6)	1 (7)
Latin America	0	1 (1)	6 (7)	4 (5)	0	0
ECOG performance status^a^, *n* (%)						
0	95 (70)	87 (66)	66 (75)	49 (59)	12 (67)	9 (64)
1	40 (30)	45 (34)	22 (25)	34 (41)	6 (33)	5 (36)
MSKCC risk group, *n* (%)						
Favourable	55 (41)	60 (45)	40 (45)	35 (42)	5 (28)	6 (43)
Intermediate	63 (47)	58 (44)	39 (44)	37 (45)	9 (50)	7 (50)
Poor	17 (13)	14 (11)	9 (10)	11 (13)	4 (22)	1 (7)
Sum of target lesion diameters, mm (range)	60.1 (0–291)	60.6 (0–231)	66.7 (0–240)	60.7 (0–217)	92.0 (0–194)	83.0 (16–190)
Metastatic sites per IRC, *n* (%)						
Lung	80 (59)	88 (67)	62 (70)	54 (65)	10 (56)	11 (79)
Liver	43 (32)	55 (42)	22 (25)	16 (19)	6 (33)	4 (29)
Bone	27 (20)	23 (17)	20 (23)	18 (22)	5 (28)	4 (29)
Lymph node	83 (61)	78 (59)	48 (55)	47 (57)	11 (61)	10 (71)
Number of prior VEGFR TKIs, *n* (%)						
1	135 (100)	132 (100)	88 (100)	83 (100)	7 (39)	8 (57)
≥2	0	0	0	0	11 (61)	6 (43)
Prior systemic therapy, *n* (%)						
Sunitinib	135 (100)	132 (100)	0	0	12 (67)	9 (64)
Pazopanib	0	0	88 (100)	83 (100)	11 (61)	6 (43)
Axitinib	0	0	0	0	5 (28)	3 (21)
Sorafenib	0	0	0	0	1 (6)	2 (14)
Bevacizumab	0	3 (2)	1 (1)	3 (4)	1 (6)	1 (7)
Interleukins	7 (5)	7 (5)	4 (5)	7 (8)	2 (11)	0
Interferons	4 (3)	7 (5)	6 (7)	5 (6)	1 (6)	0
Anti–PD- 1/PD-L1	5 (4)	5 (4)	2 (2)	3 (4)	18 (100)	14 (100)
Radiotherapy, *n* (%)	39 (29)	41 (31)	35 (40)	26 (31)	6 (33)	5 (36)
Nephrectomy, *n* (%)	116 (86)	112 (85)	76 (86)	65 (78)	16 (89)	11 (79)

*ECOG* Eastern Cooperative Oncology Group, *IRC* independent radiology committee, *MSKCC* Memorial Sloan Kettering Cancer Center, *TKI* tyrosine kinase inhibitor.

^a^Based on Karnofsky Performance Status score

As of the 31 December 2015 cut-off date for OS, the number of patients who continued to receive study treatment with cabozantinib versus everolimus was 30 (22%) versus 9 (7%) for prior sunitinib, 16 (18%) versus 7 (8%) for prior pazopanib, and 6 (33%) versus 1 (7%) for prior anti-PD-1/PD-L1.

### Efficacy outcomes

#### Number and duration of prior VEGFR TKI therapies

For patients who had received only one prior VEGFR TKI, median PFS was 7.4 months for cabozantinib versus 3.8 months for everolimus (HR 0.52, 95% CI 0.41–0.66), ORR per IRC was 17% versus 3%, and median OS was 21.4 versus 16.5 months (HR 0.65, 95% CI 0.50–0.85) **(**Supplement Table S2**)**. For patients who had received ≥2 VEGF TKIs, median PFS was 7.4 months for cabozantinib versus 4.0 months for everolimus (HR 0.51, 95% CI 0.35–0.74), ORR per IRC was 17% versus 4%, and median OS was 20.8 versus 17.2 months (HR 0.73, 95% CI 0.48–1.10).

Efficacy outcomes were also assessed based on treatment duration with first VEGFR TKI therapy. For patients previously treated ≤6 months, median PFS was 5.6 months for cabozantinib versus 3.7 months for everolimus (HR 0.62, 95% CI 0.44–0.89), ORR per IRC was 14% versus 4%, and median OS was 21.3 versus 13.8 months (HR 0.69, 95% CI 0.47–1.01) **(**Supplement Table S2**)**. For patients previously treated >6 months, median PFS was 9.0 months for cabozantinib versus 3.9 months for everolimus (HR 0.48, 95% CI 0.38–0.62), ORR per IRC was 19% versus 3%, and median OS was 22.0 versus 18.4 months (HR 0.69, 95% CI 0.52–0.90).

#### Type of prior VEGFR TKI therapy

**Fig. 1:**
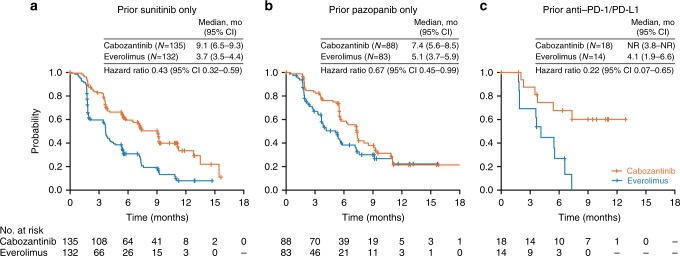
Kaplan–Meier analysis of progression-free survival. Disease progression was assessed by an independent radiology committee. Data cut-off date: 22 May 2015. CI confidence interval, NR not reached

**Fig. 2:**
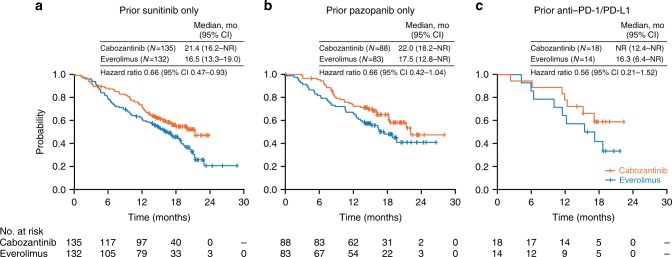
Kaplan–Meier analysis of overall survival. Data cut-off date: 31 December 2015. CI confidence interval, NR not reached

For patients treated with sunitinib as the only prior VEGFR TKI, median PFS was 9.1 months for cabozantinib versus 3.7 months for everolimus (HR 0.43, 95% CI 0.32–0.59) **(**Fig. 1**)**, ORR per IRC was 16% versus 3% **(**Table 2**)**, and median OS was 21.4 versus 16.5 months (HR 0.66, 95% CI 0.47–0.93) **(**Fig. 2**)**.

**Table 2:** Tumour response per Independent Radiology Committee

	Prior sunitinib only		Prior pazopanib only		Prior anti–PD-1/PD-L1	
	Cabozantinib *N* = 135	Everolimus *N* = 132	Cabozantinib *N* = 88	Everolimus *N* = 83	Cabozantinib *N* = 18	Everolimus *N* = 14
Objective response rate^a^ (95% CI)	16 (11–24)	3 (1–8)	19 (12–29)	4 (1–10)	22 (6–48)	0
Best overall response, *n* (%)						
Confirmed partial response	22 (16)	4 (3)	17 (19)	3 (4)	4 (22)	0
Stable disease	89 (66)	75 (57)	57 (65)	55 (66)	9 (50)	9 (64)
Progressive disease	16 (12)	46 (35)	12 (14)	18 (22)	2 (11)	4 (29)
Not evaluable or missing^b^	8 (6)	7 (5)	2 (2)	7 (8)	3 (17)	1 (7)

*CI* confidence interval.

^a^All partial responses.

^b^No qualifying post-baseline assessment for overall response

For patients treated with pazopanib as the only prior VEGFR TKI, median PFS was 7.4 months for cabozantinib versus 5.1 months for everolimus (HR 0.67, 95% CI 0.45–0.99) (Fig. 1), ORR per IRC was 19% versus 4% (Table 2), and median OS was 22.0 versus 17.5 months (HR 0.66, 95% CI 0.42–1.04) (Fig. 2).

As of 31 December 2015 cut-off date for OS, the percentage of patients who had received subsequent anticancer therapy was similar in the two treatment groups for the prior sunitinib (48% for cabozantinib versus 55% for everolimus) and prior pazopanib subgroups (52% versus 53%, respectively) (Supplement Table S3).

#### Prior therapy with Anti-PD-1/PD-L1 or IL-2

**Fig. 3:**
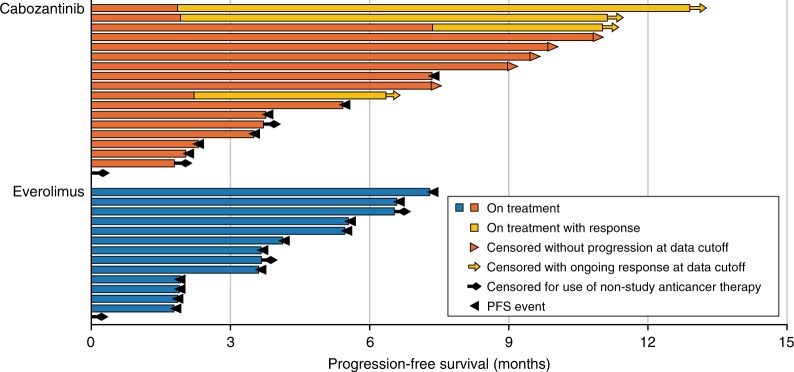
Progression-free survival and response in the prior anti-PD-1/PD-L1 subgroup. Disease progression was assessed by an independent radiology committee. Data cut-off date: 22 May 2015

Checkpoint inhibitor therapy is relatively new to the RCC treatment landscape; however, some patients enrolled in METEOR had received anti-PD-1/PD-L1 antibodies in clinical trials. For these patients, median PFS was not reached for cabozantinib compared with 4.1 months for everolimus (HR 0.22; 95% CI 0.07–0.65) (Fig. 1), and ORR per IRC was 22% versus 0% (Table 2). Median OS was not reached for cabozantinib versus 16.3 months for everolimus (HR 0.56; 95% CI 0.21–1.52) (Fig. 2). As of 22 May 2015, cut-off date for PFS, nine out of 18 patients in the cabozantinib group remained on study treatment without experiencing progression; four patients had an ongoing objective response. In the everolimus group, no patients remained on study treatment without experiencing progression (Fig. 3). As of 31 December 2015, cut-off date for OS, the percentages of patients who had received subsequent anticancer therapy were 33% in the cabozantinib group and 71% in the everolimus group (Supplement Table S3).

Efficacy outcomes were also assessed for patients who had received prior therapy with IL-2 (*N* = 49). Median PFS was 7.2 months for cabozantinib versus 5.5 months for everolimus (HR 0.57, 95% CI 0.27–1.19), ORR per IRC was 10% versus 3%, and median OS was not reached for either treatment (HR 0.75, 95% CI 0.27–2.08) (Supplement Table S4).

### Safety

The median duration of exposure was 9.2 months for cabozantinib and 4.3 months for everolimus in the prior sunitinib subgroup, 7.7 months and 4.2 months in the prior pazopanib subgroup, and 11.4 and 4.6 months in the prior anti-PD-1/PD-L1 subgroup, respectively (Supplement Table S5).

Grade 3/4 AEs were reported in 67% of cabozantinib-treated patients and 61% of everolimus-treated patients in the prior sunitinib subgroup; 73% and 62% of patients in the prior pazopanib subgroup; and 83% and 64% of patients in the prior anti-PD-1/PD-L1 subgroup (Table 3). Patients who had received sunitinib versus pazopanib as the only prior VEGFR TKI had a higher incidence (≥5% difference) of the following grade 3/4 AEs: diarrhoea (15% versus 9%), nausea (7% versus 1%), and anaemia (8% versus 3%) for cabozantinib-treated patients, and anaemia (18% versus 9%) for everolimus-treated patients. No grade 3/4 AEs were ≥5% higher in patients who had received prior pazopanib versus prior sunitinib. The incidence of AEs related to hepatotoxicity (laboratory assessments of liver function) was similar for the prior sunitinib and prior pazopanib subgroups: there were no differences ≥5% in either all grade or grade 3/4 events between the subgroups (Supplement Table S6).

**Table 3:** All causality grade 3/4 adverse events

	Prior sunitinib only		Prior pazopanib only		Prior anti–PD-1/PD-L1	
	Cabozantinib (*N* = 136)	Everolimus (*N* = 131)	Cabozantinib (*N* = 88)	Everolimus (*N* = 81)	Cabozantinib (*N* = 18)	Everolimus (*N* = 14)
Any, *n* (%)	91 (67)	80 (61)	64 (73)	50 (62)	15 (83)	9 (64)
Hypertension	22 (16)	6 (5)	14 (16)	2 (2)	4 (22)	0
Diarrhoea	21 (15)	4 (3)	8 (9)	0	2 (11)	0
Fatigue	15 (11)	9 (7)	6 (7)	8 (10)	5 (28)	2 (14)
PPE	11 (8)	0	9 (10)	2 (2)	3 (17)	1 (7)
Anaemia	11 (8)	23 (18)	3 (3)	7 (9)	2 (11)	2 (14)
Nausea	10 (7)	0	1 (1)	1 (1)	0	0
Hypomagnesemia	8 (6)	0	3 (3)	0	0	0
Hypokalaemia	8 (6)	2 (2)	2 (2)	3 (4)	0	0
Hyponatraemia	6 (4)	2 (2)	3 (3)	0	1 (6)	0
Asthaenia	5 (4)	3 (2)	2 (2)	3 (4)	2 (11)	0
Hyperglycaemia	0	6 (5)	2 (2)	3 (4)	0	0

Events that occurred at ≥ 5% frequency in either treatment arm of the overall safety population are summarised.

*PPE* palmar-plantar erythrodysesthesia

## Discussion

The phase 3 METEOR trial compared the efficacy and safety of cabozantinib with everolimus in patients with advanced RCC following VEGFR TKI therapy. Subgroup analyses reported here are consistent with results from the overall study population with observed improvements in PFS, ORR, and OS for cabozantinib compared with everolimus irrespective of prior therapy. Limitations of these analyses include the small size of the subgroups, the use of descriptive statistics, and the post hoc nature of the analyses based on prior treatment with sunitinib or pazopanib.

Historically, sequential therapy with VEGFR TKIs and mTOR inhibitors was the standard of care for advanced RCC, with sunitinib and pazopanib as preferred first-line agents and everolimus and axitinib as standard second-line therapies.^1,3,4^ The AXIS trial established axitinib as the preferred second-line VEGFR TKI based on improved PFS compared with sorafenib; however, OS was not significantly different for axitinib versus sorafenib.^14^ For the subgroup of patients who had received prior sunitinib, median PFS for axitinib was 4.8 months (HR versus sorafenib 0.74, 95% CI 0.57–0.96), and median OS was 15.2 months (HR 1.00, 95% CI 0.78–1.27).^14^

In the METEOR trial, PFS, ORR, and OS were improved for cabozantinib versus everolimus with either sunitinib or pazopanib as the only prior VEGFR TKI. These favourable results in VEGFR TKI-refractory disease support the hypothesis that the clinical activity of cabozantinib in RCC may result from combined inhibition of VEGFRs and additional targets, such as MET and AXL, that are not inhibited by other VEGFR TKIs.

Improved OS and investigator-assessed ORR have also been reported for nivolumab compared with everolimus for subgroups based on prior VEGFR TKI therapy in the CheckMate 025 trial.^15^ Median OS for nivolumab after sunitinib was 23.6 months (HR 0.81, 95% CI 0.64–1.04), and median OS for nivolumab after pazopanib was not reached (HR 0.60, 95% CI 0.42–0.84). However, subgroups in the nivolumab study were defined differently from those in current study in that sunitinib or pazopanib could have been either the first or second VEGFR TKI therapy in the CheckMate 025 analysis.

Although limited data are available regarding the efficacy of subsequent treatments for RCC following immune checkpoint therapy, VEGFR TKIs appear to have clinical activity in this setting.^16,17^ While only 5% of patients enrolled in METEOR had received prior checkpoint inhibitor therapy, outcomes for this subpopulation are clinically relevant, given the evolving role of immuno-oncology agents in RCC, including ongoing phase 3 trials in the first-line setting. In the prior anti-PD-1/PD-L1 subgroup, cabozantinib treatment was associated with improved PFS (HR 0.22, 95% CI 0.07–0.65), OS (HR 0.56, 95% CI 0.21–1.52), and ORR, as well as a higher rate of durable responses compared with everolimus. Compared with the everolimus group, patients in the cabozantinib group had less favourable MSKCC risk status, had received ≥2 prior VEGFR TKI therapies more frequently, and had received subsequent anticancer therapy less frequently. Nonetheless, cabozantinib demonstrated an apparent clinical benefit compared with everolimus in this subpopulation of third or later line patients who had received both VEGFR TKI and checkpoint inhibitor therapy. Resistance to PD-1/PD-L1 inhibition has been associated with increased expression of immunosuppressive and pro-angiogenic cytokines and transcription factors that promote epithelial-mesenchymal transition.^18,19^ Cabozantinib inhibits key mediators of both angiogenesis (VEGF receptors) and epithelial-mesenchymal transition (MET and AXL) which may partially account for its clinical activity after prior anti-PD-1/PD-L1 therapy. Moreover, cabozantinib promotes a more immune permissive tumour microenvironment,^20^ which may help overcome resistance to checkpoint inhibitors.

Current guidelines for advanced RCC recommend cabozantinib and nivolumab as second-line treatments after VEGFR-targeted therapy based on improved OS compared with everolimus; both agents are category 1 (preferred) by the National Comprehensive Cancer Network^2^ and recommended with a survival advantage by the European Association of Urology.^21^ Although no high-level evidence is available regarding subsequent therapy after either agent, subgroup analyses reported here suggest that cabozantinib is clinically active following sequential therapy with VEGFR TKIs and checkpoint inhibitors.

The types of grade 3/4 AEs reported across the prior therapy subgroups were generally consistent with those observed for the overall study population. For cabozantinib-treated patients, some events, such as diarrhoea, nausea, and anaemia, were reported at a higher rate (≥5% difference) for patients previously treated with sunitinib compared with pazopanib, suggesting that first-line VEGFR TKI therapy may influence the tolerability of subsequent treatment with cabozantinib. Although pazopanib is associated with a higher incidence of hepatoxicity than sunitinib,^22^ no difference in the incidence of AEs related to liver function was noted between the prior pazopanib and prior sunitinib subgroups. This result is consistent with previous reports that pazopanib-induced transaminitis is reversible.^23^ A higher percentage of cabozantinib-treated patients experienced grade 3/4 AEs in the prior anti-PD-1/PD-L1 subgroup compared with the overall study population (83% versus 71%).^24^ This difference may be related to the longer treatment duration in the anti-PD-1/PD-L1 subgroup compared with the overall study population (median of 11.4 versus 8.3 months), the poorer prognostic status of patients in the anti-PD-1/PD-L1 subgroup, or the small subgroup size.

Sequencing strategies for advanced RCC will continue to evolve with the approval of cabozantinib and various immuno-oncology combinations as first-line regimens. The randomised phase 2 CABOSUN trial (NCT01835158) demonstrated that cabozantinib treatment significantly improved PFS and ORR versus sunitinib in previously untreated intermediate or poor risk patients.^25^ In addition, results from the phase 3 CheckMate 214 trial (NCT02231749) demonstrated a significant improvement in OS for the combination of nivolumab and ipilimumab versus sunitinib in previously untreated patients of intermediate or poor risk.^26^ Several other phase 3 studies are exploring combinations of checkpoint inhibitors with antiangiogenic agents,^1^ including the combination of cabozantinib with nivolumab versus sunitinib (NCT03141177). With the anticipated movement of immuno-oncology combinations into the first-line setting, additional studies will be necessary to evaluate the clinical impact of subsequent therapy with targeted agents including cabozantinib.

In conclusion, cabozantinib, a second-line standard of care for advanced RCC, demonstrated improvements in PFS, ORR, and OS compared with everolimus regardless of the number, duration, or type of prior VEGFR TKI therapies. Cabozantinib treatment was also associated with improved clinical outcomes in patients who had received both VEGFR TKI therapy and an immune checkpoint inhibitor. These results highlight the broad clinical utility of cabozantinib for previously treated patients with advanced RCC, irrespective of prior therapy.

## Electronic supplementary material

Supplement

Choueiri TK, et al. Lancet Oncol. 2016:17:917–927

## References

[1] Choueiri TK, Motzer RJ (2017). Systemic therapy for metastatic renal-cell carcinoma. N. Engl. J. Med..

[2] Motzer RJ (2017). Kidney Cancer, Version 2.2017, NCCN Clinical Practice Guidelines in Oncology. J. Natl Compr. Canc. Netw..

[3] Calvo E, Schmidinger M, Heng DY, Grunwald V, Escudier B (2016). Improvement in survival end points of patients with metastatic renal cell carcinoma through sequential targeted therapy. Cancer Treat. Rev..

[4] de Velasco G, Hamieh L, Mickey S, Choueiri TK (2015). Optimizing systemic therapy for metastatic renal cell carcinoma beyond the first-line setting. Urol. Oncol..

[5] Motzer RJ (2015). Nivolumab versus everolimus in advanced renal-cell carcinoma. N. Engl. J. Med..

[6] Choueiri TK (2015). Cabozantinib versus everolimus in advanced renal-cell carcinoma. N. Engl. J. Med..

[7] Choueiri Toni K, Escudier Bernard, Powles Thomas, Tannir Nizar M, Mainwaring Paul N, Rini Brian I, Hammers Hans J, Donskov Frede, Roth Bruce J, Peltola Katriina, Lee Jae Lyun, Heng Daniel Y C, Schmidinger Manuela, Agarwal Neeraj, Sternberg Cora N, McDermott David F, Aftab Dana T, Hessel Colin, Scheffold Christian, Schwab Gisela, Hutson Thomas E, Pal Sumanta, Motzer Robert J (2016). Cabozantinib versus everolimus in advanced renal cell carcinoma (METEOR): final results from a randomised, open-label, phase 3 trial. The Lancet Oncology.

[8] Motzer RJ (2015). Lenvatinib, everolimus, and the combination in patients with metastatic renal cell carcinoma: a randomised, phase 2, open-label, multicentre trial. Lancet Oncol..

[9] Tannir NM, Schwab G, Grunwald V (2017). Cabozantinib: an Active Novel Multikinase Inhibitor in Renal Cell Carcinoma. Curr. Oncol. Rep..

[10] Yakes FM (2011). Cabozantinib (XL184), a novel MET and VEGFR2 inhibitor, simultaneously suppresses metastasis, angiogenesis, and tumor growth. Mol. Cancer Ther..

[11] Eisenhauer EA (2009). New response evaluation criteria in solid tumours: revised RECIST guideline (version 1.1). Eur. J. Cancer.

[12] Motzer RJ (2013). Prognostic factors for survival in 1059 patients treated with sunitinib for metastatic renal cell carcinoma. Br. J. Cancer.

[13] National Cancer Institute. Common Terminology Criteria for Adverse Events (CTCAE) v.4 2009. http://evs.nci.nih.gov/ftp1/CTCAE/About.html.

[14] Rini BI (2011). Comparative effectiveness of axitinib versus sorafenib in advanced renal cell carcinoma (AXIS): a randomised phase 3 trial. Lancet.

[15] Escudier Bernard, Sharma Padmanee, McDermott David F., George Saby, Hammers Hans J., Srinivas Sandhya, Tykodi Scott S., Sosman Jeffrey A., Procopio Giuseppe, Plimack Elizabeth R., Castellano Daniel, Gurney Howard, Donskov Frede, Peltola Katriina, Wagstaff John, Gauler Thomas C., Ueda Takeshi, Zhao Huanyu, Waxman Ian M., Motzer Robert J. (2017). CheckMate 025 Randomized Phase 3 Study: Outcomes by Key Baseline Factors and Prior Therapy for Nivolumab Versus Everolimus in Advanced Renal Cell Carcinoma. European Urology.

[16] Albiges L (2015). Efficacy of targeted therapies after PD-1/PD-L1 blockade in metastatic renal cell carcinoma. Eur. J. Cancer.

[17] Nadal R (2016). Safety and clinical activity of vascular endothelial growth factor receptor (VEGFR)-tyrosine kinase inhibitors after programmed cell death 1 inhibitor treatment in patients with metastatic clear cell renal cell carcinoma. Ann. Oncol..

[18] Hugo W (2016). Genomic and Transcriptomic Features of Response to Anti-PD-1 Therapy in Metastatic Melanoma. Cell.

[19] Jenkins RW, Barbie DA, Flaherty KT (2018). Mechanisms of resistance to immune checkpoint inhibitors. Br. J. Cancer.

[20] Kwilas AR, Ardiani A, Donahue RN, Aftab DT, Hodge JW (2014). Dual effects of a targeted small-molecule inhibitor (cabozantinib) on immune-mediated killing of tumor cells and immune tumor microenvironment permissiveness when combined with a cancer vaccine. J. Transl. Med..

[21] Powles T (2016). European Association of Urology Guidelines for Clear Cell Renal Cancers that are Resistant to Vascular Endothelial Growth Factor Receptor-Targeted Therapy. Eur. Urol..

[22] Motzer RJ (2013). Pazopanib versus sunitinib in metastatic renal-cell carcinoma. N. Engl. J. Med..

[23] Powles T (2015). Characterisation of liver chemistry abnormalities associated with pazopanib monotherapy: a systematic review and meta-analysis of clinical trials in advanced cancer patients. Eur. J. Cancer.

[24] Choueiri TK (2016). Cabozantinib versus everolimus in advanced renal cell carcinoma (METEOR): final results from a randomised, open-label, phase 3 trial. Lancet Oncol..

[25] Choueiri TK (2017). Cabozantinib versus sunitinib as initial targeted therapy for patients with metastatic renal cell carcinoma of poor or intermediate risk: the Alliance A031203 CABOSUN Trial. J. Clin. Oncol..

[26] Escudier B. et al. CheckMate 214: efficacy and safety of nivolumab + ipilimumab (N + I) v sunitinib (S) for treatment-naïve advanced or metastatic renal cell carcinoma (mRCC), including IMDC risk and PD-L1 expression subgroups. Abstract (LBA5) presented at: Annual Congress of the European Society for Medical Oncology; September 8–12, 2017; Madrid, Spain. 2017.

