# Supplementary material for: Outcomes based on prior therapy in the phase 3 METEOR trial of cabozantinib versus everolimus in advanced renal cell carcinoma
Source: Br J Cancer. 2018 Sep 10;119(6):663–9. doi: 10.1038/s41416-018-0164-0 (PMC6173766; doi:10.1038/s41416-018-0164-0)
Supplement: Supplementary file 1 — Supplement [file 41416_2018_164_MOESM1_ESM.docx]

**Online Only Supplement**

**Table S1. Prior Therapy Subgroups……..…………………………………………………………………...…2**

**Table S2. Efficacy by Number of Prior VEGFR TKIs and Duration of Treatment with First VEGFR TKI……………………………………………………………………………………………………………………..3**

**Table S3. Subsequent Anticancer Therapy……………………………………………...………………….....4**

**Table S4. Efficacy for Prior IL-2 Subgroup……………………………………………......…...…….….….....5**

**Table S5. Duration of Exposure…….……………………………………………………………………………5**

**Table S6. Adverse Events for Laboratory Assessments of Liver Function……………………………...6**

**Figure S1. Patient disposition as of December, 31 2015, in the subgroups of patients who received sunitinib (A) or pazopanib (B) as their only prior VEGFR TKI, or prior therapy with a PD-1/PD-L1 immune checkpoint inhibitor (C)………..……………………………………….………………………………7**

**Table S1. Prior Therapy Subgroups**

| **Prior Anticancer Regimens, n (%)** | **Cabozantinib**  **(N=330)** | **Everolimus**  **(N=328)** |
| --- | --- | --- |
| 1 prior VEGFR TKI | 235 (71) | 229 (70) |
| Prior Sunitinib only | 135 (41) | 132 (40) |
| Prior Pazopanib only | 88 (27) | 83 (25) |
| ≥2 prior VEGFR TKIs | 95 (29) | 99 (30) |
| Duration of treatment with first VEGFR TKI^a^ | | |
| ≤6 months | 88 (27) | 102 (31) |
| >6 months | 242 (73) | 224 (68) |
| Prior anti-PD-1/PD-L1^b^ | 18 (5) | 14 (4) |
| Prior IL-2 | 20 (6) | 29 (9) |

^a^Treatment duration missing for two patients in everolimus arm; ^b^Thirty-one patients received nivolumab, one patient in the cabozantinib arm received atezolizumab.

TKI, tyrosine kinase inhibitor.

**Table S2. Efficacy by Number of Prior VEGFR TKIs and Duration of Treatment with First VEGFR TKI**

| **Number of prior VEGFR TKIs** | **1 VEGFR TKI** | | | **≥2 VEGFR TKIs** | | |
| --- | --- | --- | --- | --- | --- | --- |
|  | **Cabozantinib**  **N=235** | **Everolimus**  **N=229** | **HR (95% CI)** | **Cabozantinib**  **N=95** | **Everolimus**  **N=99** | **HR (95% CI)** |
| Median PFS, mo (95% CI) | 7.4 (6.5–9.2) | 3.8 (3.7–5.1) | 0.52 (0.41–0.66) | 7.4 (5.6–9.2) | 4.0 (3.7–5.6) | 0.51 (0.35–0.74) |
| Median OS, mo (95% CI) | 21.4 (18.4–NR) | 16.5 (14.0–18.9) | 0.65 (0.50–0.85) | 20.8 (16.1–NR) | 17.2 (13.8–NR) | 0.73 (0.48–1.10) |
| ORR per IRC, % (95% CI) | 17 (13–23) | 3 (1–6) |  | 17 (10–26) | 4 (1–10) |  |
|  |  | | |  | | |
| **Treatment duration with first VEGFR TKI^a^** | **≤6 months** | | | **>6 months** | | |
|  | **Cabozantinib**  **N=88** | **Everolimus**  **N=102** | **HR (95% CI)** | **Cabozantinib**  **N=242** | **Everolimus**  **N=224** | **HR (95% CI)** |
| Median PFS, mo (95% CI) | 5.6 (3.8–7.4) | 3.7 (2.8–5.4) | 0.62 (0.44–0.89) | 9.0 (7.3–9.2) | 3.9 (3.7–5.4) | 0.48 (0.38–0.62) |
| Median OS, mo (95% CI) | 21.3 (12.4–NR) | 13.8 (10.6–16.4) | 0.69 (0.47–1.01) | 22.0 (18.7–NR) | 18.4 (16.1–20.8) | 0.69 (0.52–0.90) |
| ORR per IRC, % (95% CI) | 14 (7–23) | 4 (1–10) |  | 19 (14–24) | 3 (1–6) |  |

^a^Treatment duration missing for 2 patients in the everolimus arm.
CI, confidence interval; HR, hazard ratio; IRC, independent radiology committee; NR, not reached; ORR, objective response rate; OS, overall survival; PFS, progressive-free survival; TKI, tyrosine kinase inhibitor.

**Table S3. Subsequent Anticancer Therapy**

| **Subsequent Anticancer Regimens, n (%)** | **Prior Sunitinib Only** | | **Prior Pazopanib Only** | | **Prior Anti-PD-1/PD-L1** | |
| --- | --- | --- | --- | --- | --- | --- |
|  | **Cabozantinib**  **(N=135)** | **Everolimus**  **(N=132)** | **Cabozantinib**  **(N=88)** | **Everolimus**  **(N=83)** | **Cabozantinib**  **(N=18)** | **Everolimus**  **(N=14)** |
| Any systemic anticancer therapy | 65 (48) | 73 (55) | 46 (52) | 44 (53) | 6 (33) | 10 (71) |
| Any VEGFR TKI | 33 (24) | 61 (46) | 22 (25) | 43 (52) | 4 (22) | 10 (71) |
| Axitinib | 24 (18) | 51 (39) | 18 (20) | 27 (33) | 3 (17) | 7 (50) |
| Sorafenib | 3 (2) | 9 (7) | 2 (2) | 10 (12) | 0 | 3 (21) |
| Sunitinib | 3 (2) | 4 (3) | 7 (8) | 11 (13) | 2 (11) | 1 (7) |
| Pazopanib | 4 (3) | 9 (7) | 0 | 1 (1) | 0 | 2 (14) |
| Cabozantinib | 0 | 3 (2) | 0 | 2 (2) | 0 | 0 |
| Anti-PD-1/PD-L1/PD-L2 | 3 (2) | 5 (4) | 5 (6) | 5 (6) | 0 | 0 |
| Everolimus | 34 (25) | 5 (4) | 28 (32) | 2 (2) | 2 (11) | 0 |
| Temsirolimus | 2 (1) | 2 (2) | 0 | 1 (1) | 1 (6) | 1 (7) |
| Chemotherapy | 2 (1) | 6 (5) | 2 (2) | 2 (2) | 1 (6) | 0 |
| Bevacizumab | 3 (2) | 3 (2) | 4 (5) | 5 (6) | 1 (6) | 0 |
| Interleukins | 0 | 2 (2) | 0 | 1 (1) | 0 | 1 (7) |
| Interferons | 3 (2) | 5 (4) | 1 (1) | 0 | 0 | 0 |

TKI, tyrosine kinase inhibitor.

**Table S4. Efficacy for Prior IL-2 Subgroup**

|  |  | | |
| --- | --- | --- | --- |
|  | **Cabozantinib**  **N=20** | **Everolimus**  **N=29** | **HR (95% CI)** |
| Median PFS, mo (95% CI) | 7.2 (3.8–11.2) | 5.5 (3.5–5.6) | 0.57 (0.27–1.19) |
| Median OS, mo (95% CI) | NR (17.1–NR) | NR (15.0–NR) | 0.75 (0.27–2.08) |
| ORR per IRC, % (95% CI) | 10 (1–32) | 3 (0–17) |  |

**Table S5. Duration of Exposure**

|  | **Prior Sunitinib Only** | | **Prior Pazopanib Only** | | **Prior Anti-PD-1/PD-L1** | |
| --- | --- | --- | --- | --- | --- | --- |
|  | **Cabozantinib**  **(N=136)** | **Everolimus**  **(N=131)** | **Cabozantinib**  **(N=88)** | **Everolimus**  **(N=81)** | **Cabozantinib**  **(N=18)** | **Everolimus**  **(N=14)** |
| Median duration of exposure, mo (IQR) | 9.2  (4.5–14.5) | 4.3  (1.9–8.3) | 7.7  (4.2–14.5) | 4.2  (1.9–8.8) | 11.4  (2.8–17.3) | 4.6  (2.8–5.7) |

IQR, interquartile range.

**Table S6. Adverse Events for Laboratory Assessments of Liver Function**

| **Adverse Event** | **Prior Sunitinib Only** | | | | **Prior Pazopanib Only** | | | |
| --- | --- | --- | --- | --- | --- | --- | --- | --- |
|  | **Cabozantinib**  **(N=136)** | | **Everolimus**  **(N=131)** | | **Cabozantinib**  **(N=88)** | | **Everolimus**  **(N=81)** | |
|  | Any Grade | Grade 3/4 | Any Grade | Grade 3/4 | Any Grade | Grade 3/4 | Any Grade | Grade 3/4 |
| Aspartate aminotransferase increased | 24 (18) | 2 (1) | 6 (5) | 0 | 13 (15) | 1 (1) | 4 (5) | 0 |
| Alanine aminotransferase increased | 19 (14) | 3 (2) | 7 (5) | 1 (1) | 15 (17) | 2 (2) | 5 (6) | 0 |
| Gamma-glutamyltransferase increased | 7 (5) | 2 (1) | 10 (8) | 6 (5) | 7 (8) | 2 (2) | 4 (5) | 1 (1) |
| Bilirubin increased | 5 (4) | 4 (3) | 0 | 0 | 3 (3) | 1 (1) | 0 | 0 |
| Alkaline phosphatase increased | 6 (4) | 1 (1) | 6 (5) | 1 (1) | 4 (5) | 0 | 4 (5) | 0 |
| Hypoalbuminaemia | 9 (7) | 0 | 2 (2) | 0 | 4 (5) | 1 (1) | 2 (2) | 0 |

**Figure S1. Patient disposition as of December, 31 2015, in the subgroups of patients who received sunitinib (A) or pazopanib (B) as their only prior VEGFR TKI, or prior therapy with a PD-1/PD-L1 immune checkpoint inhibitor (C)**

TKI, tyrosine kinase inhibitor.

Disposition for the overall population has been published: Choueiri TK, et al. Lancet Oncol. 2016;17(7):917-927.
