# Supplementary material for: Outcomes based on prior therapy in the phase 3 METEOR trial of cabozantinib versus everolimus in advanced renal cell carcinoma
Source: Br J Cancer. 2018 Sep 10;119(6):663–9. doi: 10.1038/s41416-018-0164-0 (PMC6173766; doi:10.1038/s41416-018-0164-0)
Supplement: Supplementary file 2 — Choueiri TK, et al. Lancet Oncol. 2016:17:917–927 [file 41416_2018_164_MOESM2_ESM.pdf]

Cabozantinib versus everolimus in advanced renal cell carcinoma (METEOR): final results from a randomised, open-label, phase 3 trial

Toni K Choueiri, Bernard Escudier, Thomas Powles, Nizar M Tannir, Paul N Mainwaring, Brian I Rini, Hans J Hammers, Frede Donskov, Bruce J Roth, Katriina Peltola, Jae Lyun Lee, Daniel Y C Heng, Manuela Schmidinger, Neeraj Agarwal, Cora N Sternberg, David F McDermott, Dana T Aftab, Colin Hessel, Christian Scheffold, Gisela Schwab, Thomas E Hutson, Sumanta Pal, Robert J Motzer, for the METEOR investigators

Summary

Background Cabozantinib is an oral inhibitor of tyrosine kinases including MET, VEGFR, and AXL. The randomised phase 3 METEOR trial compared the efficacy and safety of cabozantinib versus the mTOR inhibitor everolimus in patients with advanced renal cell carcinoma who progressed after previous VEGFR tyrosine-kinase inhibitor treatment. Here, we report the final overall survival results from this study based on an unplanned second interim analysis.

Methods In this open-label, randomised phase 3 trial, we randomly assigned (1:1) patients aged 18 years and older with advanced or metastatic clear-cell renal cell carcinoma, measurable disease, and previous treatment with one or more VEGFR tyrosine-kinase inhibitors to receive 60 mg cabozantinib once a day or 10 mg everolimus once a day. Randomisation was done with an interactive voice and web response system. Stratification factors were Memorial Sloan Kettering Cancer Center risk group and the number of previous treatments with VEGFR tyrosine-kinase inhibitors. The primary endpoint was progression-free survival as assessed by an independent radiology review committee in the first 375 randomly assigned patients and has been previously reported. Secondary endpoints were overall survival and objective response in all randomly assigned patients assessed by intention-to-treat. Safety was assessed per protocol in all patients who received at least one dose of study drug. The study is closed for enrolment but treatment and follow-up of patients is ongoing for long-term safety evaluation. This trial is registered with ClinicalTrials.gov, number NCT01865747.

Findings Between Aug 8, 2013, and Nov 24, 2014, 658 patients were randomly assigned to receive cabozantinib (n=330) or everolimus (n=328). The median duration of follow-up for overall survival and safety was 18·7 months (IQR 16·1–21·1) in the cabozantinib group and 18·8 months (16·0–21·2) in the everolimus group. Median overall survival was 21·4 months (95% CI 18·7–not estimable) with cabozantinib and 16·5 months (14·7–18·8) with everolimus (hazard ratio [HR] 0·66 [95% CI 0·53–0·83]; p=0·00026). Cabozantinib treatment also resulted in improved progression-free survival (HR 0·51 [95% CI 0·41–0·62]; p<0·0001) and objective response (17% [13–22] with cabozantinib *vs* 3% [2–6] with everolimus; p<0·0001) per independent radiology review among all randomised patients. The most common grade 3 or 4 adverse events were hypertension (49 [15%] in the cabozantinib group *vs* 12 [4%] in the everolimus group), diarrhoea (43 [13%] *vs* 7 [2%]), fatigue (36 [11%] *vs* 24 [7%]), palmar-plantar erythrodysaesthesia syndrome (27 [8%] *vs* 3 [1%]), anaemia (19 [6%] *vs* 53 [17%]), hyperglycaemia (3 [1%] *vs* 16 [5%]), and hypomagnesaemia (16 [5%] *vs* none). Serious adverse events grade 3 or worse occurred in 130 (39%) patients in the cabozantinib group and in 129 (40%) in the everolimus group. One treatment-related death occurred in the cabozantinib group (death; not otherwise specified) and two occurred in the everolimus group (one aspergillus infection and one pneumonia aspiration).

Interpretation Treatment with cabozantinib increased overall survival, delayed disease progression, and improved the objective response compared with everolimus. Based on these results, cabozantinib should be considered as a new standard-of-care treatment option for previously treated patients with advanced renal cell carcinoma. Patients should be monitored for adverse events that might require dose modifications.

Funding Exelixis Inc.

Introduction

Advances in the understanding of the molecular pathology of renal cell carcinoma have led to the development of agents targeting the VEGFR and mTOR signalling pathways. First-line standard-of-care treatments for patients with advanced renal cell carcinoma are the VEGFR tyrosine-kinase inhibitors sunitinib and pazopanib. Second-line standard-of-care treatments include the VEGFR tyrosine-kinase inhibitors axitinib and sorafenib, the mTOR inhibitor everolimus, and the PD-1 checkpoint inhibitor nivolumab.[1–3] Few treatments have shown a survival benefit, and none have shown an improvement in all three efficacy endpoints of progression-free survival, objective response, and overall survival, compared with standard-of-care treatment in a randomised phase 3 trial in previously treated patients with renal cell carcinoma.

Lancet Oncol 2016

Published Online
June 5, 2016
http://dx.doi.org/10.1016/S1470-2045(16)30107-3

See Online/Comment
http://dx.doi.org/10.1016/S1470-2045(16)30175-9

Dana–Farber Cancer Institute, Boston, MA, USA (T K Choueiri MD); **Institut Gustave Roussy, Villejuif, France** (B Escudier MD); **Barts Cancer Institute, Cancer Research UK Experimental Cancer Medicine Centre, Queen Mary University of London, Royal Free NHS Trust, London, UK** (Prof T Powles MD); **University of Texas, MD Anderson Cancer Center Hospital, Houston, TX, USA** (Prof N M Tannir MD); **Icon Cancer Care, Brisbane, Queensland, Australia** (P N Mainwaring MD); **Cleveland Clinic Foundation, Cleveland, OH, USA** (Prof B I Rini MD); **Johns Hopkins Sidney Kimmel Comprehensive Cancer Center, Baltimore, MD, USA** (H J Hammers MD); **Aarhus University Hospital, Aarhus, Denmark** (F Donskov MD); **Washington University in St Louis, St Louis, MO, USA** (Prof B J Roth MD); **Helsinki University Central Hospital Cancer Center, Helsinki, Finland** (K Peltola MD); **Asan Medical Center, University of Ulsan College of Medicine, Seoul, South Korea** (Prof J Lyun Lee MD); **Tom Baker Cancer Centre, University of Calgary, Calgary, Alberta, Canada** (D Y C Heng MD); **Medical University of Vienna, Vienna, Austria** (Prof M Schmidinger MD); **Huntsman Cancer Institute at The University of Utah, Salt Lake City, Utah** (N Agarwal MD); **San Camillo and Forlanini Hospitals, Rome, Italy** (Prof C N Sternberg MD); **Beth**

Israel Deaconess Medical Center, Boston, MA, USA (D F McDermott MD); **Exelixis, South San Francisco, CA, USA** (D T Aftab PhD, C Hessel MS, C Scheffold MD, G Schwab MD); **Texas Oncology–Baylor Sammons Cancer Center, Dallas, TX, USA** (Prof T E Hutson DO); **City of Hope National Medical Center, Duarte, CA, USA** (S Pal MD); **and Memorial Sloan Kettering Cancer Center, New York, NY, USA** (Prof R J Motzer MD)

Correspondence to: Dr Toni K Choueiri, Dana-Farber Cancer Institute, Boston, MA, USA **toni_choueiri@dfci.harvard.edu**

Research in context

Evidence before this study

In the randomised, phase 3 METEOR trial, treatment with cabozantinib, an inhibitor of tyrosine kinases including MET, VEGFR, and AXL, significantly improved progression-free survival compared with treatment with everolimus in previously treated patients with advanced renal cell carcinoma. In a scientific literature review up to March 10, 2016, we searched PubMed with the search terms, phase 3, overall survival, ORR, PFS, RCC, AXL, MET, VEGFR, and TKI. None of the currently approved treatments had shown significant benefit for all three efficacy endpoints of overall survival, progression-free survival, and objective response in a pivotal phase 3 trial in previously treated patients with advanced renal cell carcinoma. We also found data suggesting that increased expression of MET and AXL are associated with a poor prognosis in renal cell carcinoma patients, and that inhibition of these targets may help to overcome resistance to VEGF pathway inhibition.

Added value of this study

Our findings show that treatment with cabozantinib was associated with a significant increase in overall survival, in addition to an improvement in progression-free survival and objective response, when compared with treatment with everolimus, a second-line standard of care in patients with second-line advanced renal cell carcinoma. The recorded clinical benefits were consistent in all subgroups. The safety profile of cabozantinib was consistent with that previously reported.

Implications of all the available evidence

The improvements in progression-free survival, overall survival, and objective response suggest that cabozantinib should be considered as a new treatment for previously treated patients with advanced renal cell carcinoma. Recently, the immune checkpoint inhibitor nivolumab also improved overall survival compared with everolimus in this population, but without improving progression-free survival. Future research on the optimal use of cabozantinib and other available treatments might help to provide maximum benefit to patients with advanced renal cell carcinoma.

Cabozantinib is an oral inhibitor of tyrosine kinases including MET, VEGFR, and AXL.[4] Upregulation of MET and AXL in clear-cell renal cell carcinoma happens as a consequence of von Hippel-Lindau protein dysfunction, has been implicated in tumour progression and VEGFR tyrosine-kinase inhibitor resistance in preclinical studies, and has been associated with a poor prognosis in patients with renal cell carcinoma.[5–10] The randomised phase 3 METEOR trial[11] compared the efficacy and safety of cabozantinib versus the mTOR inhibitor everolimus in patients with advanced renal cell carcinoma who progressed after previous VEGFR tyrosine-kinase inhibitor treatment. Progression-free survival in the first 375 randomised patients, the primary endpoint, was significantly improved with cabozantinib compared with everolimus treatment with a median progression-free survival of 7·4 months (95% CI 5·6–9·1) versus 3·8 months (3·7–5·4; HR 0·58, 95% CI 0·45–0·75; p<0·001) as assessed by an independent radiology review committee.[11]

Here we report the final overall survival results from the METEOR study based on an unplanned second interim analysis. Analyses of progression-free survival and objective response in all randomised patients, and updated safety data are also reported.

Methods

Study design and participants

METEOR is a randomised, open-label, phase 3, study with patients enrolled at 173 hospital and outpatient clinics in 26 countries (appendix pp 2–5).[11] Patients 18 years or older with advanced or metastatic renal cell carcinoma and a clear-cell histology were eligible for enrolment if they had measurable disease per Response Evaluation Criteria in Solid Tumors (RECIST version 1.1),[12] had received at least one previous VEGFR tyrosine-kinase inhibitor (there was no limit to the number of previous treatments), and had disease progression during or within 6 months of the most recent VEGFR tyrosine-kinase inhibitor treatment and within 6 months before randomisation. Patients were required to have a Karnofsky performance status score of at least 70% and adequate organ function, based on standard laboratory tests including haematology, serum chemistry, lipids, coagulation, thyroid function, and urinalysis. Patients with brain metastases were allowed provided these were stable and asymptomatic. Patients with previous mTOR inhibitor therapy, including everolimus, were not eligible for the study nor were patients with uncontrolled hypertension or clinically significant cardiovascular, gastrointestinal, wound healing, or infectious comorbidities. The study adhered to the Good Clinical Practice guidelines and the Declaration of Helsinki. The institutional review board or ethics committee of the participating centres approved the study protocol. All patients provided written informed consent.

See **Online** for appendix

Randomisation and masking

Patients were randomly assigned (1:1) to receive either cabozantinib or everolimus. Randomisation was stratified by the number of previous VEGFR tyrosine-kinase inhibitor treatments (1 or ≥2) and Memorial Sloan Kettering Cancer Center (MSKCC) risk group (favourable, intermediate, or poor) for previously treated patients[13] (appendix p 6). We used stratified permuted blocks as the

randomisation schema. Study treatment was assigned centrally with an interactive voice and web response system. Study personnel did not have access to the master list of blocks or block sizes. Patients and investigators were not masked to study treatment to allow appropriate management of adverse events. Aggregate summaries of efficacy data by treatment group were not done until the time of the primary progression-free survival analysis.

Procedures

Cabozantinib was given orally once a day at 60 mg and everolimus was given orally once a day at 10 mg. Everolimus was chosen as the comparator for cabozantinib since it is an accepted standard-of-care in second-line therapy for advanced renal cell carcinoma.[1,14] Treatment modifications, including interruptions and dose reductions, were specified to manage adverse events. Cabozantinib could be dose reduced to 40 mg and then 20 mg, and everolimus could be dose reduced to 5 mg and then 2·5 mg. Patients were allowed to continue study treatment beyond radiographic progression at the discretion of the investigator. On-study crossover between treatment groups was not permitted. Safety evaluations including physical examination, vital signs, laboratory tests, and adverse event assessments were done every 2 weeks for the first 8 weeks and then every 4 weeks thereafter. ECG assessments were performed every 4 weeks for the first 8 weeks and then every 12 weeks. A safety follow-up visit was scheduled 30 days after treatment discontinuation. Adverse events were assessed by investigators and graded according to Common Terminology Criteria for Adverse Events (CTCAE version 4.0).[15]

Radiographic assessments by CT or MRI were done at screening and every 8 weeks for the first 12 months and then every 12 weeks thereafter. Tumour response and progression were assessed according to RECIST (version 1.1)[12] by a masked centralised independent radiology review committee. Patients were followed for overall survival every 8 weeks.

Tumour tissue (archival or recently biopsied) was obtained at enrolment when available for immunohistochemistry analysis of MET protein levels. Formalin-fixed paraffin embedded tumour blocks or freshly cut formalin-fixed paraffin embedded slides were analysed by LabCorp (Research Triangle Park, NC, USA) with the SP44 antibody (Spring Biosciences, Pleasanton, CA, USA). MET expression was defined as high versus low, based on a cutoff of 50% or higher of tumour tissue stained with an intensity of 2+ or 3+ by immunohistochemistry, according to published procedures.[16,17]

Outcomes

The primary endpoint was progression-free survival by independent radiology review in the first 375 randomised patients. Progression-free survival was defined as the time from randomisation to radiographic progression per RECIST or death from any cause. The secondary endpoints were overall survival, defined as the time from randomisation to death from any cause, and objective response per independent radiology review committee assessment, defined as the proportion of patients with a confirmed complete or partial response per RECIST, assessed in all randomly assigned patients. Safety and tolerability were also assessed.

Statistical analysis

The study was designed to provide adequate power for both progression-free survival and overall survival analyses. For the primary endpoint of progression-free survival, the event-driven analysis (at the two-sided 5% α level) required 259 progression-free survival events. The secondary endpoints of overall survival (at the two-sided 4% α level) and objective response (at the two-sided 1% α level) were to be tested in all randomly assigned patients (per the intention-to-treat principle) at the time of the primary progression-free survival analysis only if the primary progression-free survival endpoint was significant. For overall survival, assuming one interim analysis at the time of the primary endpoint analysis and a subsequent final analysis, 408 deaths were required to provide 80% power to detect a hypothesised HR of 0·75 corresponding to an improvement in median survival from 15 months[18] to 20 months. With a planned average accrual rate of 32 patients per month and using a 1:1 treatment allocation ratio, 650 patients were required to observe 408 deaths within the planned study duration of 36 months. As the total sample size of 650 required to evaluate overall survival was much larger than needed to assess the primary endpoint of progression-free survival there was the possibility that patients with earlier onset of radiographic progression would be over-represented (and those with later onset of radiographic progression under-represented) among the planned 259 progression-free survival events. To reduce this potential bias, the primary analysis of progression-free survival was prespecified to occur when the required 259 events were observed in the first 375 randomised patients, the size the study would have been without the overall survival endpoint. Supportive analyses of progression-free survival among all randomly assigned patients were also planned.

The planned interim analysis of overall survival (done at the time of the primary progression-free survival analysis with a data cutoff of May 22, 2015; minimum follow-up of 6 months) at that time did not meet the boundary for significance (HR 0·67, 95% CI 0·51–0·89; p=0·005; 49% information fraction: critical p value ≤0·0019) defined by the Lan-DeMets O'Brien-Fleming alpha spending function. The results of the planned interim analysis of overall survival were made public in July, 2015, and were published in September, 2015.[11] The decision to conduct an unplanned second interim

analysis was made by the funder in consultation with regulatory agencies. As a result, the analysis plan was revised in October, 2015, to include an unplanned second interim analysis of overall survival with a prospectively defined cutoff date of Dec 31, 2015, to provide a minimum of 13 months of follow-up from the last patient enrolled. At this analysis, the critical p value to achieve significance from the alpha spending function was 0·0163 or lower.

We did hypothesis testing of overall survival and progression-free survival with the stratified log-rank test with the randomisation stratification factors. Median duration of progression-free survival and overall survival, corresponding 95% confidence intervals, and landmark proportions were estimated by the Kaplan-Meier method. Hazard ratios (HRs) were estimated with a Cox regression model adjusted for the randomisation stratification factors. The proportional hazards assumption was evaluated by visual inspection of log-log plots. A post-hoc sensitivity analysis of progression-free survival per independent radiology review committee among the 283 patients randomised after the first 375 was conducted using the same methods as the primary analysis. Post-hoc analysis of patients who continued on study treatment for at least 2 weeks after radiographic progression as determined by the investigator evaluated post-progression changes in tumour status by two criteria: the proportion with at least one assessment of stable disease or partial response (from randomisation) after progression; and the proportion with at least one assessment in which the sum of target lesion diameters was lower than the pre-randomisation baseline value. Hypothesis testing for objective response was done with the Cochran-Mantel-Haenzel method, and confidence intervals for proportions were calculated by the Clopper-Pearson method. All subgroup analyses of progression-free survival and overall survival were prespecified except for the subgroups based on receiving sunitinib or pazopanib as the only previous VEGFR tyrosine-kinase inhibitor. ECOG performance status was converted from Karnofsky status using ECOG 0 for Karnofsky status of 100% and 90% as ECOG 1 for Karnofsky status of 80% and 70%. Confidence intervals and p values for subgroup analyses are considered descriptive. HRs reported for subgroup analyses are unadjusted. Safety analyses were limited to patients who received any amount of study treatment and analysed per protocol. All analyses were done with SAS (version 9.1 or higher).

This trial is registered at ClinicalTrials.gov, number NCT01865747.

Role of the funding source

The funder was involved in the study design, data collection, and analysis. The authors and the funder were involved in data interpretation. The steering committee members (TKC, BE, TP, and RJM) and DTA, CHH, CS, and GS had access to the raw data. The first draft of the manuscript was written by TKC and RJM in collaboration with the funder. Medical writing support and cabozantinib

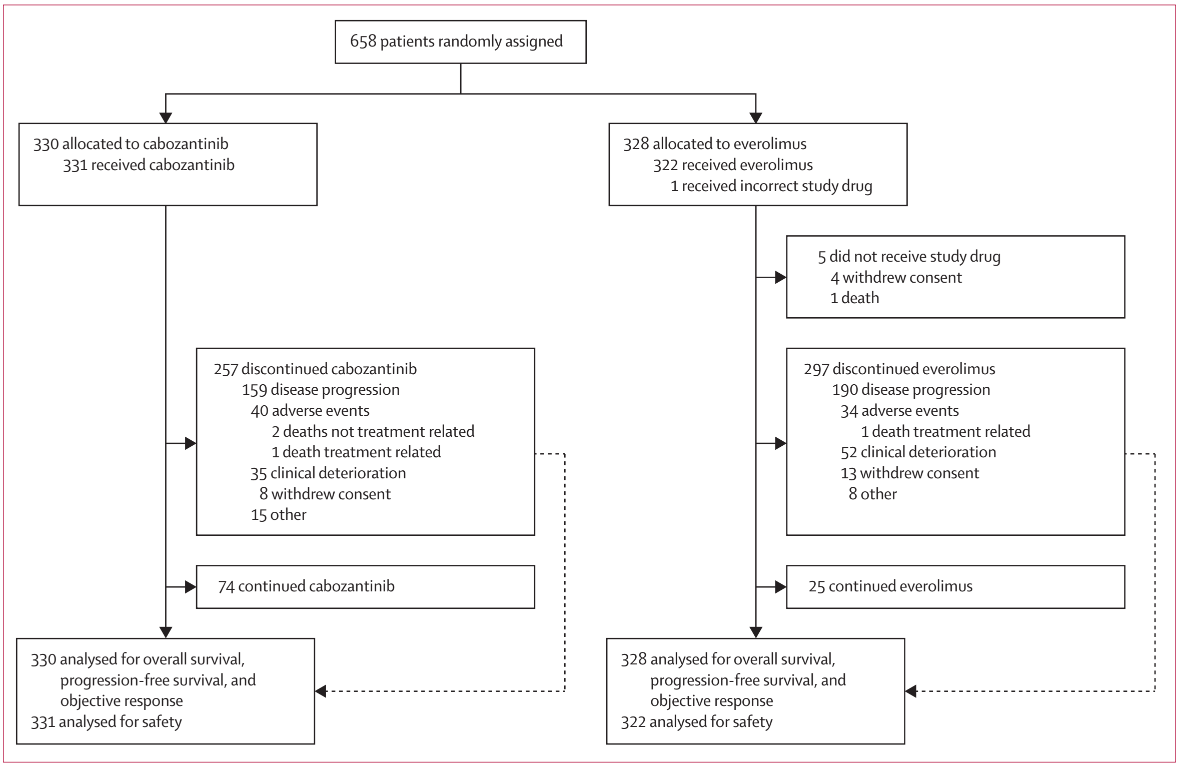

Figure 1: **Trial profile of the final analysis of overall survival**

and everolimus were provided by the funder. All authors gave final approval for submission and the corresponding author had the final responsibility to submit for publication.

Results

Between Aug 8, 2013, and Nov 24, 2014, 658 patients were randomly assigned to receive cabozantinib (n=330) or everolimus (n=328; figure 1). Demographics and baseline characteristics were typical of patients with advanced renal cell carcinoma and were balanced between treatment groups (table 1).[11]

As of Dec 31, 2015, the cutoff for the unplanned second interim analysis of overall survival, 74 (22%) of 330 patients in the cabozantinib group and 25 (8%) of 328 patients in the everolimus group remained on study treatment. The median duration of follow-up for overall survival and safety was 18·7 months (IQR 16·1–21·1) in the cabozantinib group and 18·8 months (16·0–21·2) in the everolimus group. The second interim analysis of overall survival included 320 deaths, representing 78% of the 408 deaths planned for the prespecified final analysis of overall survival: 140 (42%) patients died in the cabozantinib group and 180 (55%) died in the everolimus group. Survival status as of the cutoff date was determined for most (98%) of the 658 randomly assigned patients.

Treatment with cabozantinib significantly increased overall survival compared with that in patients treated with everolimus (figure 2). The median overall survival was 21·4 months (95% CI 18·7–not estimable) in the cabozantinib group compared with 16·5 months (14·7–18·8) in the everolimus group. The HR was 0·66 (95% CI 0·53–0·83; p=0·00026), which met the criterion for significance (p≤0·0163) from the prespecified alpha spending function. A log-log plot of the survivor functions (appendix p 6) had reasonably parallel lines, supporting the assumption of proportional hazards.

Kaplan-Meier landmark estimates at 6, 12, 18, and 24 months showed that at each timepoint the proportion of patients estimated to be alive was greater in the cabozantinib group compared with the everolimus group (appendix p 6). All subgroup analyses of overall survival were consistent with the results for the overall population (figure 3). The appendix shows Kaplan-Meier plots of overall survival for patients with high and low tumour MET expression status (p 7).

The data cutoff for progression-free survival and objective response analyses in all randomised patients was the same as for the primary progression-free survival analysis (May 22, 2015) with a median duration of follow-up of 11·4 months (IQR 8·8–13·7) in the cabozantinib group and 11·5 months (8·6–13·9) in the everolimus group.[11] 180 events occurred in the cabozantinib group and 214 in the everolimus group. The analysis of progression-free survival per independent radiology review done in all 658 randomly assigned patients showed improved progression-free survival with

	Cabozantinib (N=330)	Everolimus (N=328)
Age (years)	63 (56–68)	62 (55–68)
Sex		
Male	253 (77%)	241 (73%)
Female	77 (23%)	86 (26%)
Not reported	0	1 (<1%)
Geographic region		
Europe	167 (51%)	153 (47%)
North America	118 (36%)	122 (37%)
Asia–Pacific	39 (12%)	47 (14%)
Latin America	6 (2%)	6 (2%)
Race		
White	269 (82%)	263 (80%)
Asian	21 (6%)	26 (8%)
Black	6 (2%)	3 (<1%)
Other	19 (6%)	13 (4%)
Not reported	15 (5%)	22 (7%)
Missing data	0	1 (<1)
ECOG performance status score		
0	226 (68%)	217 (66%)
1	104 (32%)	111 (34%)
MSKCC prognostic risk category		
Favourable	150 (45%)	150 (46%)
Intermediate	139 (42%)	135 (41%)
Poor	41 (12%)	43 (13%)
Metastatic site per IRC		
Lung	204 (62%)	212 (65%)
Liver	88 (27%)	103 (31%)
Bone	77 (23%)	65 (20%)
Lymph node	206 (62%)	199 (61%)
Brain	2 (<1%)	1 (<1%)
Other	23 (7%)	21 (6%)
Sum of target lesion diameters per IRC (mm)	65 (37–105)	65 (41–111)
Previous VEGFR tyrosine-kinase inhibitors		
1	235 (71%)	229 (70%)
≥2	95 (29%)	99 (30%)
Previous systemic therapy		
Sunitinib	210 (64%)	205 (62%)
Pazopanib	144 (44%)	136 (41%)
Axitinib	52 (16%)	55 (17%)
Sorafenib	21 (6%)	31 (9%)
Bevacizumab	5 (2%)	11 (3%)
Interleukin 2	20 (6%)	29 (9%)
Interferon α	19 (6%)	24 (7%)
Nivolumab*	17 (5%)	14 (4%)
Radiotherapy	110 (33%)	108 (33%)
Nephrectomy	283 (86%)	279 (85%)

Data are n (%) or median (IQR). ECOG=Eastern Cooperative Oncology Group. IRC=independent radiology review committee. MSKCC=Memorial Sloan Kettering Cancer Center. *One additional patient in the cabozantinib group received prior atezolizumab.

Table 1: **Demographics and baseline characteristics**

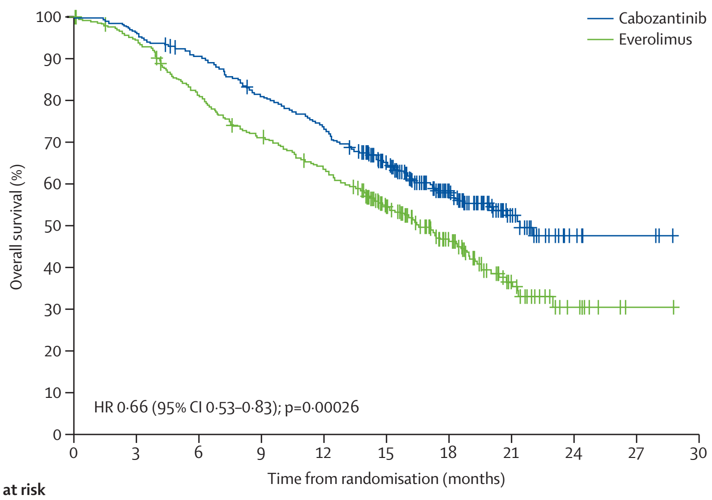

Number at risk	0	3	6	9	12	15	18	21	24	27	30
Cabozantinib	330	318	296	264	239	178	105	41	6	3	0
Everolimus	328	307	262	229	202	141	82	32	8	1	0
**Number censored**											
Cabozantinib	0	0	3	1	0	35	57	56	32	3	3
Everolimus	0	3	2	1	2	34	42	36	20	7	1

***Figure 2:* Kaplan-Meier plot of overall survival through Dec 31, 2015**
All 658 randomly assigned patients were included in the analysis. The number of patients censored is summarised by interval. HR=hazard ratio.

cabozantinib compared with everolimus (HR 0·51 [95% CI 0·41–0·62]; p<0·0001; figure 4). The median progression-free survival was 7·4 months (95% CI 6·6–9·1) in the cabozantinib group versus 3·9 months (3·7–5·1) in the everolimus group. These results were consistent with the previously reported primary endpoint of progression-free survival done in the first 375 randomly assigned patients[11] and an additional post-hoc sensitivity analysis of progression-free survival per independent radiology review done in the 283 patients not included in the primary progression-free survival analysis (HR 0·44 [95% CI 0·31–0·61]). Results for progression-free survival per investigator assessment were similar to those shown by the independent radiology review committee (appendix p 9). Subgroup analyses of progression-free survival per independent radiology review were also consistent with the results for the overall population (figure 3).

The proportion of patients who achieved an objective response per independent radiology review in all 658 randomly assigned patients was 57 (17% [95% CI 13–22]; 57 partial responses) of 330 in the cabozantinib group and 11 (3% [2–6]; 11 partial responses) of 328 in the everolimus group (p<0·0001; appendix p 9). Results for tumour response per investigator assessment were similar to those established by the independent radiology review committee (appendix p 9).

As of the May 22, 2015, cutoff date, the proportions of patients continuing study treatment for at least 2 weeks after radiographic progression as assessed by the investigator were similar between groups, 74 (38%) of 193 who progressed on cabozantinib and 71 (31%) of 226 who progressed on everolimus). Post-hoc analyses of response for these patients showed that five (7%) of 74 patients in the cabozantinib group and six (8%) of 71 in the everolimus group had stable disease or a partial response after the initial radiographic progression. Additionally, 34 (46%) of 74 patients in the cabozantinib group and 15 (21%) of 71 in the everolimus group had at least one assessment in which the sum of target lesion diameters was lower than the pre-randomisation baseline value.

As of the Dec 31, 2015, the cutoff for the overall survival analysis, the median duration of exposure was 8·3 months (IQR 4·2–14·6) in patients given cabozantinib (n=331) and 4·4 months (1·9–8·6) in patients given everolimus (n=322). Dose reductions occurred for 206 (62%) patients in the cabozantinib group and 80 (25%) patients in the everolimus group. The median daily dose was 43 mg (IQR 36–56) cabozantinib and 9 mg (7–10) everolimus. Treatment discontinuation because of an adverse event not related to disease progression was recorded in 40 (12%) of 331 patients in the cabozantinib group and 34 (11%) of 322 patients in the everolimus group. The most frequent reason for treatment discontinuation in both groups was disease progression (figure 1). Similar proportions of patients in the everolimus group and the cabozantinib group were reported to have received subsequent systemic anticancer treatment after study treatment discontinuation (181 [55%] *vs* 165 [50%]; appendix p 8).

The overall incidence of adverse events irrespective of causality was 100% for both groups (331 of 331 patients treated with cabozantinib and 321 of 322 treated with everolimus). We recorded grade 3 or 4 adverse events in 235 (71%) patients treated with cabozantinib and 193 (60%) treated with everolimus (table 2; appendix pp 10–15). The most common grade 3 or 4 adverse events were hypertension (49 [15%] in the cabozantinib group *vs* 12 [4%] in the everolimus group), diarrhoea (43 [13%] *vs* 7 [2%]), fatigue (36 [11%] *vs* 24 [7%]), palmar-plantar erythrodysaesthesia syndrome (27 [8%] *vs* 3 [1%]), anaemia (19 [6%] *vs* 53 [17%]), hyperglycaemia (3 [1%] *vs* 16 [5%]), and hypomagnesaemia (16 [5%] *vs* none).

Grade 3 or worse serious adverse events occurred in 130 (39%) patients in the cabozantinib group and in 129 (40%) in the everolimus group. The most common grade 3 or worse serious adverse events were abdominal pain (nine [3%] in the cabozantinib group *vs* three [1%] in the everolimus group), pleural effusion (eight [2%] *vs* seven [2%]), pneumonia (seven [2%] *vs* 13 [4%]), pulmonary embolism (seven [2%] *vs* one [<1%], anaemia (five [2%] *vs* 10 [3%]), and dyspnoea (four [1%] *vs* 10 [3%]). Deaths during the adverse events reporting period, irrespective of causality, occurred in 26 patients (8%) in the cabozantinib group and 25 (8%) in the everolimus group; most of these were related to disease progression

	Patients cabozantinib/everolimus	Overall survival: Events cabozantinib/everolimus	Overall survival: HR (95% CI)	Progression-free survival: Events cabozantinib/everolimus	Progression-free survival: HR (95% CI)
**Overall**	**330/328**	**140/180**	**0·66 (0·53–0·83)**	**180/214**	**0·51 (0·41–0·62)**
Age					
<65 years	196/198	86/107	0·72 (0·54–0·95)	109/133	0·53 (0·41–0·68)
≥65 years	134/130	54/73	0·62 (0·44–0·88)	71/81	0·50 (0·36–0·69)
Sex					
Female	77/86	37/53	0·74 (0·48–1·12)	48/55	0·72 (0·49–1·07)
Male	253/241	103/126	0·66 (0·51–0·85)	132/158	0·46 (0·36–0·58)
Race					
Non-white	46/42	22/25	0·65 (0·37–1·15)	27/28	0·49 (0·28–0·83)
White	269/263	110/143	0·65 (0·51–0·83)	142/169	0·50 (0·40–0·63)
MSKCC risk group					
Favourable	150/150	48/66	0·66 (0·46–0·96)	79/92	0·51 (0·38–0·69)
Intermediate	139/135	64/79	0·67 (0·48–0·94)	74/89	0·47 (0·35–0·65)
Poor	41/43	28/35	0·65 (0·39–1·07)	27/33	0·70 (0·42–1·16)
IMDC risk group					
Favourable	66/62	14/17	0·70 (0·34–1·41)	34/37	0·47 (0·30–0·76)
Intermediate	210/214	89/121	0·65 (0·49–0·85)	107/137	0·48 (0·37–0·62)
Poor	54/52	37/42	0·74 (0·48–1·15)	39/40	0·67 (0·48–1·04)
Previous nephrectomy					
No	47/49	24/32	0·75 (0·44–1·27)	25/32	0·51 (0·30–0·86)
Yes	283/279	116/148	0·66 (0·52–0·84)	155/182	0·51 (0·41–0·64)
ECOG status					
0	226/216	81/105	0·65 (0·49–0·87)	114/137	0·46 (0·36–0·59)
1	104/112	59/75	0·72 (0·51–1·02)	66/77	0·64 (0·46–0·90)
Diagnosis to randomisation					
<1 year	59/76	35/52	0·89 (0·58–1·37)	37/57	0·55 (0·36–0·84)
≥1 year	271/251	105/127	0·66 (0·51–0·85)	143/156	0·51 (0·41–0·65)
Tumour MET status					
High	51/50	20/27	0·55 (0·31–0·99)	26/36	0·41 (0·24–0·68)
Low	150/162	63/87	0·72 (0·52–1·00)	79/97	0·58 (0·43–0·79)
Unknown	129/116	57/66	0·67 (0·47–0·95)	75/81	0·50 (0·36–0·68)
Number of organs with metastases					
1	59/56	18/24	0·72 (0·39–1·34)	33/32	0·84 (0·52–1·37)
2	101/77	38/37	0·73 (0·47–1·16)	52/48	0·60 (0·40–0·89)
≥3	168/190	84/117	0·65 (0·49–0·86)	95/132	0·38 (0·29–0·50)
SoD					
<median	165/163	57/72	0·76 (0·54–1·08)	87/98	0·63 (0·47–0·84)
≥median	165/164	83/107	0·60 (0·45–0·80)	93/115	0·41 (0·31–0·54)
Bone metastases					
No	253/263	105/137	0·71 (0·55–0·91)	140/169	0·57 (0·45–0·71)
Yes	77/65	35/43	0·54 (0·34–0·84)	40/45	0·33 (0·21–0·51)
Visceral metastases					
No	89/83	32/40	0·70 (0·44–1·12)	44/47	0·64 (0·42–0·97)
Yes	241/245	108/140	0·66 (0·52–0·85)	136/167	0·48 (0·38–0·60)
Visceral and bone metastases					
No	270/276	111/140	0·73 (0·57–0·93)	145/175	0·56 (0·45–0·70)
Yes	60/52	29/40	0·45 (0·28–0·72)	35/39	0·26 (0·16–0·43)
Number of previous VEGFR TKIs					
1	235/229	98/130	0·65 (0·50–0·85)	131/155	0·52 (0·41–0·66)
≥2	95/99	42/50	0·73 (0·48–1·10)	49/59	0·51 (0·35–0·74)
Duration of first VEGFR TKI					
≤6 months	88/102	42/65	0·69 (0·47–1·01)	56/70	0·62 (0·44–0·89)
>6 months	242/224	98/114	0·69 (0·52–0·90)	124/142	0·48 (0·38–0·62)
Progression after start of most recent VEGFR TKI					
<3 months	44/68	25/45	0·76 (0·47–1·24)	29/47	0·67 (0·42–1·07)
≥3 months	283/259	113/134	0·68 (0·53–0·88)	148/166	0·50 (0·40–0·62)
Previous PD-1 or PD-L1 treatment					
No	312/314	133/171	0·68 (0·54–0·85)	174/203	0·54 (0·44–0·66)
Yes	18/14	7/9	0·56 (0·21–1·52)	6/11	0·22 (0·07–0·65)
Only previous VEGFR TKI					
Sunitinib	135/132	59/80	0·66 (0·47–0·93)	74/97	0·43 (0·32–0·59)
Pazopanib	88/83	34/42	0·66 (0·42–1·04)	51/49	0·67 (0·45–0·99)

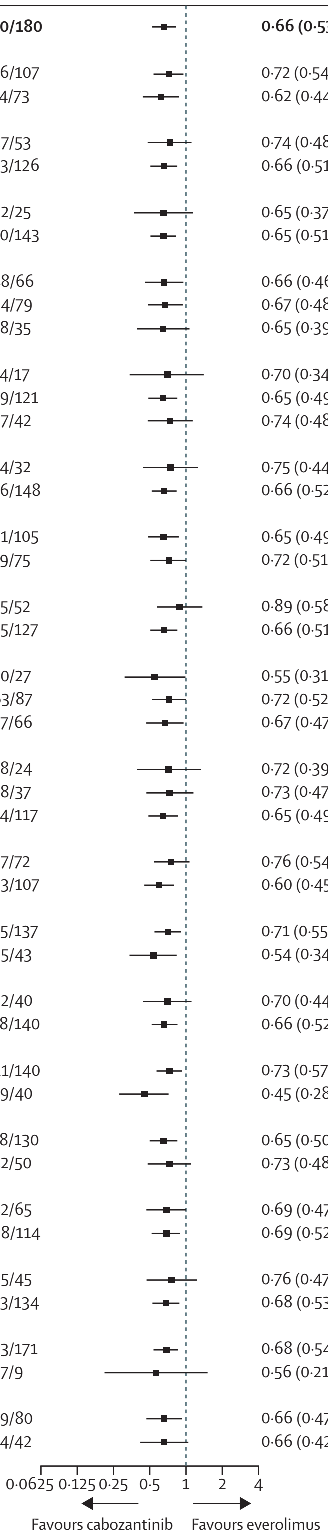

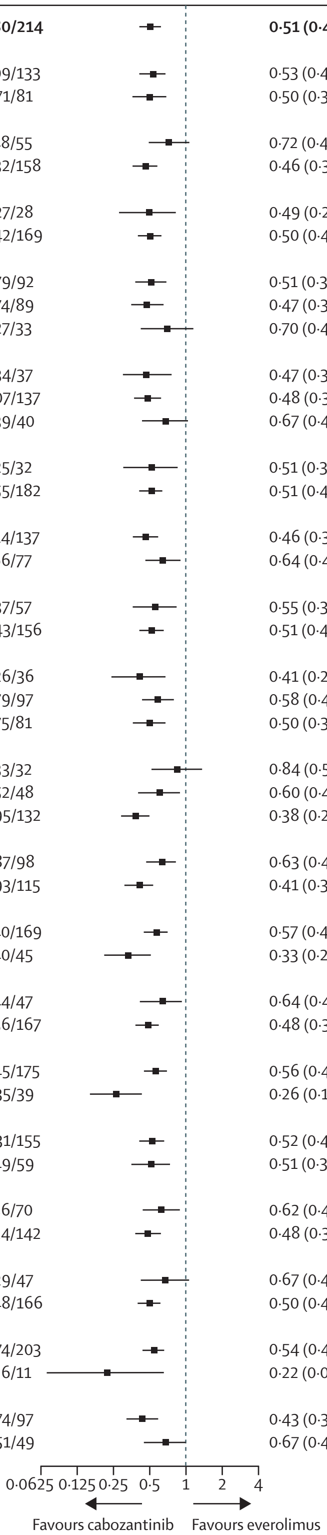

***Figure 3:* Forest plots of overall survival and progression-free survival**
All 658 randomly assigned patients were included in the analyses of overall survival (data cutoff of Dec 31, 2015) and progression-free survival (data cutoff of May 22, 2015). Disease progression and metastatic sites were assessed by an independent radiology review committee. Hazard ratios are estimates from the Cox proportional hazards model and are unstratified with the exception of those for the overall population, which use the stratification factors for randomisation. The available MET data differ between the overall survival and progression-free survival analyses. For the progression-free survival analyses by MET status, the following tumour MET data were available for the cabozantinib group versus the everolimus group: MET high (48 patients *vs* 48 patients), MET low (138 *vs* 151), and unknown MET status (144 *vs* 129). HR=hazard ratio. MSKCC=Memorial Sloan Kettering Cancer Center. IMDC=International Metastatic Renal Cell Carcinoma Database Consortium.[18] ECOG=Eastern Cooperative Oncology Group. SoD=sum of target lesion diameters. TKI=tyrosine-kinase inhibitor.

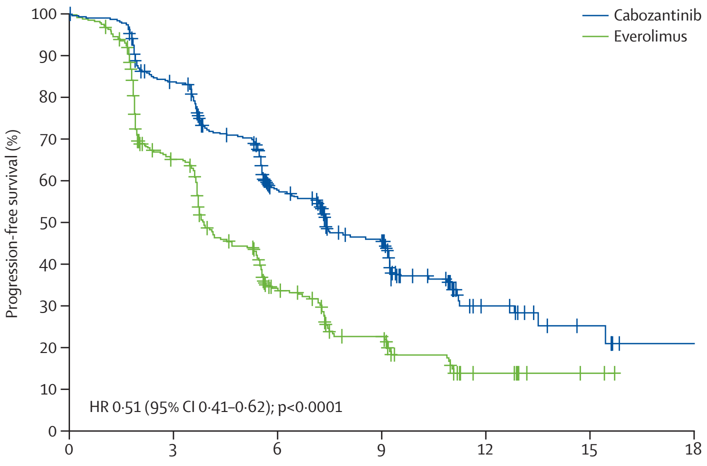

	0	3	6	9	12	15	18
**Number at risk**							
Cabozantinib	330	261	148	88	20	6	2
Everolimus	328	174	72	37	10	2	0
**Number censored**							
Cabozantinib	0	17	37	32	47	12	3
Everolimus	0	51	24	13	16	8	2

Figure 4: **Kaplan-Meier plot of progression-free survival**
Data are as of May 22, 2015. Disease progression was assessed by an independent radiology review committee in all 658 randomly assigned patients. The number of patients censored is summarised by interval. HR=hazard ratio.

(appendix p 10). One death was assessed as treatment related in the cabozantinib group (death; not otherwise specified) and two were assessed as treatment-related in the everolimus group (one aspergillus infection and one pneumonia aspiration).

Discussion

Our findings showed a significant overall survival benefit for cabozantinib treatment compared with everolimus treatment in patients with previously treated advanced renal cell carcinoma. Progression-free survival was also significantly improved in the cabozantinib group versus the everolimus group and we recorded a higher proportion of patients with an objective response in the cabozantinib group compared with the everolimus group. The progression-free survival results in all randomly assigned patients were consistent with the results previously reported for the first 375 randomly assigned patients (the primary endpoint of the study).[11] Objective tumour response per independent radiology review was also consistent with the previously reported results for the first 375 randomly assigned patients.

The updated safety profile of cabozantinib was similar to that previously reported at the earlier data cutoff for this study.[11] The most common adverse events were typical of those recorded with other VEGFR tyrosine-kinase inhibitors in patients with renal cell carcinoma.[19] The adverse event profile for everolimus was similar to that reported in other renal cell carcinoma studies.[20] Adverse events were managed with dose modifications and supportive care in both treatment groups. Although the frequency of dose reductions was higher in the cabozantinib group, the number of treatment discontinuations due to adverse events was similar in both groups, suggesting that dose modifications were effective in minimising or preventing treatment-associated discontinuations.

The results for overall survival and progression-free survival in all subgroups were consistent with those for the overall population, including those defined by the prespecified stratification factors (MSKCC risk group and number of previous VEGFR tyrosine-kinase inhibitors). The most common previous treatments were sunitinib and pazopanib, consistent with standard clinical practice.[1] Outcomes for patients refractory to first-line VEGFR tyrosine-kinase inhibitor treatment are not available because this information was not collected. The effects of cabozantinib on overall survival and progression-free survival in patients with bone metastases, which are associated with a poor prognosis,[21] are consistent with reported effects of cabozantinib on bone metastases in both clinical and preclinical studies,[22,23] and warrant further investigation into the mechanisms underlying the activity of cabozantinib in bone.

The proportions of patients continuing study treatment for at least 2 weeks after radiographic progression were similar between groups. The proportions of patients who received subsequent anticancer treatment after study treatment discontinuation were also similar between the treatment groups. Therefore, these factors are unlikely to have biased the results of overall survival towards one treatment group. Additionally, treatment crossover was not allowed after determination of the primary endpoint of progression-free survival, enabling robust assessment of overall survival.

Although the study used an open-label design, bias was minimised for the primary endpoint of progression-free survival and secondary endpoint of objective response by evaluation of radiographic assessments by a masked central independent radiology review committee. Additionally, radiographic assessments were continued beyond investigator-determined progression to reduce missing data arising from discordance between the investigator and the independent radiology review committee about the date of progression. An advantage of open-label design is appropriate management of adverse effects in both study groups.

High MET expression in patients with advanced renal cell carcinoma has been associated with both a poor prognosis and prior exposure to VEGFR tyrosine-kinase inhibitors, and in preclinical models is associated with resistance to VEGFR tyrosine-kinase inhibitor treatment.[5,8,9,24] Therefore, because cabozantinib targets receptor tyrosine kinases including MET, we investigated MET expression by immunohistochemistry as a potentially predictive biomarker for cabozantinib in this study population. However, the results suggest that the MET expression level might not affect treatment

 www.thelancet.com/oncology **Published online June 5, 2016** **http://dx.doi.org/10.1016/S1470-2045(16)30107-3**

outcomes with cabozantinib in this patient population, which might reflect the broader target profile of cabozantinib. A limitation of this analysis was that archival tumour tissue was used in most cases rather than a fresh biopsy obtained before study treatment initiation, which could have resulted in MET expression values that were not contemporaneous with the disease state during study treatment. Additionally, about one third of randomly assigned patients had an unknown MET status because archival tumour tissue was not available.

Our results support the hypothesis that the target profile of cabozantinib, which inhibits MET and AXL in addition to VEGF receptors, might help to overcome resistance to VEGFR inhibition.[5–10] This view is supported by the low incidence of refractory disease recorded, with only 12% of patients experiencing progressive disease as a best response with cabozantinib treatment. Furthermore, the effects of cabozantinib were consistently noted irrespective of the duration of the first previous VEGFR tyrosine-kinase inhibitor treatment. The results also suggest that sequenced VEGFR inhibition can be beneficial for the treatment of advanced renal cell carcinoma, which has also been suggested by the report of a phase 2 study with levantinib.[25] Additional studies are necessary to clearly define the roles of MET, AXL, and other targets beyond VEGFR in the clinical activity recorded with cabozantinib in patients with advanced renal cell carcinoma. Future studies could also include more detailed assessment of response to first-line therapy and tumour biopsies at the time of progression to better define the mechanisms of resistance and the possible benefits of sequenced VEGFR inhibition.

Several VEGFR tyrosine-kinase inhibitors have previously been approved for the treatment of metastatic renal cell carcinoma, and these comprise the mainstay of present day treatment. Regulatory approval for each of these (sunitinib, sorafenib, pazopanib, and axitinib) was based on an improvement in progression-free survival over control groups of cytokine (interferon), placebo, or sorafenib in a randomised phase 3 trial.[26–29] The mTOR inhibitor everolimus, which was used as the comparator arm in this study, was also approved based on improved progression-free survival when compared to placebo.[30] None of the pivotal phase 3 trials for these previously approved agents showed a significant benefit in overall survival. Improvement in overall survival remains the gold standard as an endpoint for representing clinical benefit in patients. It is therefore notable that in this study cabozantinib treatment resulted in a benefit in overall survival in addition to improved progression-free survival and objective response compared with everolimus in patients who had progressed on standard VEGFR tyrosine-kinase inhibitor treatments. The difference in overall survival between cabozantinib and everolimus highlights the clinical activity of cabozantinib in advanced renal cell carcinoma.

	Cabozantinib (N=331)			Everolimus (N=322)		
	Grade 1–2	Grade 3	Grade 4	Grade 1–2	Grade 3	Grade 4
Any adverse event	70 (21%)	210 (63%)	25 (8%)	103 (32%)	167 (52%)	26 (8%)
Diarrhoea	206 (62%)	43 (13%)	0	85 (26%)	7 (2%)	0
Fatigue	159 (48%)	36 (11%)	0	130 (40%)	24 (7%)	0
Nausea	158 (48%)	15 (5%)	0	92 (29%)	1 (<1%)	0
Decreased appetite	146 (44%)	10 (3%)	0	111 (35%)	3 (1%)	0
Palmar-plantar erythrodysaesthesia syndrome	115 (35%)	27 (8%)	0	16 (5%)	3 (1%)	0
Vomiting	106 (32%)	7 (2%)	0	44 (14%)	3 (1%)	0
Weight decreased	105 (32%)	9 (3%)	0	42 (13%)	0	0
Constipation	89 (27%)	1 (<1%)	0	64 (20%)	1 (<1%)	0
Dysgeusia	80 (24%)	0	0	30 (9%)	0	0
Hypothyroidism	76 (23%)	0	0	1 (<1%)	1 (<1%)	0
Hypertension	73 (22%)	49 (15%)	0	14 (4%)	12 (4%)	0
Dysphonia	68 (21%)	2 (1%)	0	16 (5%)	0	0
Cough	67 (20%)	1 (<1%)	0	107 (33%)	3 (1%)	0
Stomatitis	65 (20%)	8 (2%)	0	71 (22%)	7 (2%)	0
Mucosal inflammation	60 (18%)	5 (2%)	0	64 (20%)	10 (3%)	1 (<1%)
Dyspnoea	56 (17%)	10 (3%)	0	82 (26%)	11 (3%)	3 (1%)
Aspartate aminotransferase increased	55 (17%)	5 (2%)	0	19 (6%)	1 (<1%)	0
Back pain	54 (16%)	8 (2%)	0	41 (13%)	7 (2%)	0
Rash	52 (16%)	2 (1%)	0	92 (29%)	2 (1%)	0
Asthenia	49 (15%)	15 (5%)	0	46 (14%)	8 (2%)	0
Abdominal pain	48 (15%)	12 (4%)	0	27 (8%)	5 (2%)	0
Alanine aminotransferase increased	47 (14%)	7 (2%)	1 (<1%)	20 (6%)	1 (<1%)	0
Pain in extremity	46 (14%)	5 (2%)	0	31 (10%)	1 (<1%)	0
Muscle spasms	45 (14%)	0	0	17 (5%)	0	0
Arthralgia	43 (13%)	1 (<1%)	0	46 (14%)	4 (1%)	0
Headache	43 (13%)	1 (<1%)	0	42 (13%)	1 (<1%)	0
Anaemia	42 (13%)	19 (6%)	0	73 (23%)	53 (17%)	0
Dizziness	41 (12%)	1 (<1%)	0	21 (7%)	0	0
Dyspepsia	40 (12%)	1 (<1%)	0	15 (5%)	0	0
Oedema peripheral	39 (12%)	0	0	70 (22%)	6 (2%)	0
Hypomagnesaemia	38 (12%)	6 (2%)	10 (3%)	5 (2%)	0	0
Dry skin	37 (11%)	0	0	35 (11%)	0	0
Proteinuria	37 (11%)	8 (2%)	0	28 (9%)	2 (1%)	0
Flatulence	33 (10%)	0	0	7 (2%)	0	0
Insomnia	32 (10%)	0	0	33 (10%)	1 (<1%)	0
Pyrexia	31 (9%)	3 (1%)	0	57 (18%)	2 (1%)	0
Pruritus	27 (8%)	0	0	48 (15%)	1 (<1%)	0
Blood creatinine increased	17 (5%)	1 (<1%)	0	39 (12%)	0	0
Hypertriglyceridaemia	17 (5%)	4 (1%)	0	31 (10%)	7 (2%)	3 (1%)
Hyperglycaemia	15 (5%)	2 (1%)	1 (<1%)	46 (14%)	16 (5%)	0
Epistaxis	14 (4%)	0	0	46 (14%)	0	0

Adverse events that were reported as grade 1–2 in at least 10% of the patients in either study group are shown, irrespective of whether the event was considered by the investigator to be related to the study treatment. All grade 3, 4, and 5 events are listed in the appendix (p 10). One treatment-related death occurred in the cabozantinib group (death; not otherwise specified) and two occurred in the everolimus group (one aspergillus infection and one pneumonia aspiration). Patients are counted once at the highest grade for each preferred term. The severity of adverse events was graded according to the National Cancer Institute Common Terminology Criteria for Adverse Events (version 4.0).

Table 2: **Adverse events**

The immune checkpoint inhibitor nivolumab has been shown to have an overall survival benefit with an improved objective response compared with everolimus after previous antiangiogenic treatment for renal cell carcinoma, but no progression-free survival benefit was recorded.[3] The HR for overall survival in the nivolumab study was similar to the HR with cabozantinib in this study (0·73 *vs* 0·66, both compared with everolimus); however, the median overall survival of the everolimus group differed (19·6 months in the nivolumab study and 16·5 months in this study), reflecting the challenges of cross-trial comparison. The limits of publicly available data make it difficult to identify any factors to explain the difference in overall survival in the control groups of the two studies. The recorded median overall survival for patients treated with everolimus in our study was consistent with that reported in the pivotal RECORD-1 study (14·8 months).[20]

In this randomised phase 3 study, treatment with cabozantinib was associated with clinically significant improvements in overall survival, progression-free survival, and objective response compared with treatment with everolimus, a standard of care in second-line advanced renal cell carcinoma. The observed clinical activity of cabozantinib was applicable to patients in all risk categories and irrespective of previous treatments and the extent of tumour burden. Based on these results, cabozantinib should be considered a new treatment option for previously treated patients with advanced renal cell carcinoma.

Contributors

TKC, BE, TP, RJM, DTA, CH, CS, and GS were involved in study and protocol design. TKC was the study chair. Patients were recruited by all authors except DTA, CH, CS, and GS. The study statistician was CH. All authors contributed to data collection, interpretation, and writing of the report. All authors approved the final draft.

Declaration of interests

DTA is an employee and stockholder of Exelixis Inc and has various patents related to cabozantinib that are pending or issued. NA has received a consultancy fee for participation in advisory boards from Pfizer, Argos, Cerulean, Exelixis, and Medivation and NA's institution has received grants for the submitted work from Exelixis for conduct of this trial. TKC has received consultancy fees from Pfizer, Bayer, Novartis, GSK, Merck, Bristol-Myers Squibb, Roche, Eisai, Prometheus Labs Inc, Foundation Medicine Inc, and Cerulean. TKC's institution has received grants for research funding from Pfizer, Novartis, Merck, Exelixis, Tracon, GSK, Bristol-Myers Squibb, AstraZeneca, Peloton, and Roche. FD has received funding for study expenses from Exelixis during the conduct of this study. HJH has received clinical trial support from Exelixis during the conduct of this study. DYCH has received consultancy fees from Exelixis, Novartis, Pfizer, and Bristol-Myers Squibb and has received a grant for the submitted work from Exelixis. CH is an employee and stockholder of Exelixis. TEH has received consultancy fees and speaker honoraria from Pfizer, Novartis, Eisai, Exelixis, Genentech, Bristol-Myers Squibb, and AVEO. PNM reports consultancy fees for participation in advisory boards and speaker honoraria from Astellas and Janssen. DFM has received consultancy fee for participation in advisory boards from Exelixis, Novartis, Bristol Myers Squibb, Merck, and Genentech, and consultancy fee for participation in data and safety monitoring boards from Pfizer. RNM has received consultancy fees from Pfizer, Novartis, and Eisai; and his institution has received grants for trial support from Pfizer, Novartis, Eisai, Bristol-Myers Squibb, and Genentech/Roche. SP has received consultancy fees from Exelixis during the conduct of this study. KP has received advisory fees from Lilly, Novartis, Pfizer, Sanofi, Astellas, and Merck Sharp and Dohme; advisory fees and travel compensations from Bristol-Myers Squibb; travel compensation from Pierre-Fabre; and has received part-time salary from Orion Pharma. TP has received grants from Exelixis, Novartis, and Roche during the conduct of this study. He has received personal fees from Novartis, Pfizer, and Roche during the conduct of this study. BIR has received grants and personal fees from Exelixis. BJR's institution has received grants for the submitted work from Exelixis for conduct of this trial (no personal reimbursement). CSc is an employee and stockholder of Exelixis. MS has received speaker honoraria from Pfizer, Roche, Bristol-Myers Squibb, and Novartis; consultancy fees from Pfizer, Roche, Novartis, Bristol-Myers Squibb, and Exelixis; and research funding from Pfizer and Roche. GS is an employee and stockholder of Exelixis. CNS has received speaker honoraria from Novartis, GSK, Pfizer, and Eisai. NMT reports grants, personal fees and non-financial support from Novartis, Bristol-Myers Squibb, and Exelixis; personal fees and non-financial support from GSK, Pfizer, and Nektar; and grants from Epizyme. All other authors declare no competing interests.

Acknowledgments

We thank the patients, their families, the investigators and site staff, and the study teams who participated in the METEOR trial. This study was funded by Exelixis Inc. Medical writing support was provided by Julie C Lougheed (Exelixis), and editorial support was provided by Michael Raffin (Fishawack Communications, Horsham, PA, USA), which was funded by Exelixis.

References

1 Motzer RJ, Jonasch E, Agarwal N, et al. Kidney cancer, version 3. 2015. *J Natl Compr Canc Netw* 2015; **13:** 151–59.

2 Powles T, Staehler M, Ljungberg B, et al. Updated EAU guidelines for clear cell renal cancer patients who fail VEGF targeted therapy. *Eur Urol* 2016; **69:** 4–6.

3 Motzer RJ, Escudier B, McDermott DF, et al. Nivolumab versus everolimus in advanced renal-cell carcinoma. *N Engl J Med* 2015; **373:** 1803–13.

4 Yakes FM, Chen J, Tan J, et al. Cabozantinib (XL184), a novel MET and VEGFR2 inhibitor, simultaneously suppresses metastasis, angiogenesis, and tumor growth. *Mol Cancer Ther* 2011; **10:** 2298–308.

5 Gibney GT, Aziz SA, Camp RL, et al. c-Met is a prognostic marker and potential therapeutic target in clear cell renal cell carcinoma. *Ann Oncol* 2013; **24:** 343–49.

6 Gustafsson A, Martuszewska D, Johansson M, et al. Differential expression of Axl and Gas6 in renal cell carcinoma reflecting tumor advancement and survival. *Clin Cancer Res* 2009; **15:** 4742–49.

7 Rankin EB, Fuh KC, Castellini L, et al. Direct regulation of GAS6/AXL signaling by HIF promotes renal metastasis through SRC and MET. *Proc Natl Acad Sci U S A* 2014; **111:** 13373–78.

8 Zhou L, Liu XD, Sun M, et al. Targeting MET and AXL overcomes resistance to sunitinib therapy in renal cell carcinoma. *Oncogene* 2015 Sept 14. DOI:10.1038/onc.2015.343.

9 Harshman LC, Choueiri TK. Targeting the hepatocyte growth factor/c-Met signaling pathway in renal cell carcinoma. *Cancer J* 2013; **19:** 316–23.

10 Pinato DJ, Chowdhury S, Stebbing J. TAMing resistance to multi-targeted kinase inhibitors through Axl and Met inhibition. *Oncogene* 2015 Oct 5. DOI:10.1038/onc.2015.374.

11 Choueiri TK, Escudier B, Powles T, et al. Cabozantinib versus Everolimus in Advanced Renal-Cell Carcinoma. *N Engl J Med* 2015; **373:** 1814–23.

12 Eisenhauer EA, Therasse P, Bogaerts J, et al. New response evaluation criteria in solid tumours: revised RECIST guideline (version 1.1). *Eur J Cancer* 2009; **45:** 228–47.

13 Motzer RJ, Bacik J, Schwartz LH, et al. Prognostic factors for survival in previously treated patients with metastatic renal cell carcinoma. *J Clin Oncol* 2004; **22:** 454–63.

14 Ljungberg B, Bensalah K, Canfield S, et al. EAU guidelines on renal cell carcinoma: 2014 update. *Eur Urol* 2015; **67:** 913–24.

15 National Cancer Institute. Common Terminology Criteria for Adverse Events (CTCAE) version 4. http://evs.nci.nih.gov/ftp1/CTCAE/About.html (accessed Jan 28, 2015).

16 Spigel DR, Ervin TJ, Ramlau RA, et al. Randomized phase II trial of Onartuzumab in combination with erlotinib in patients with advanced non-small-cell lung cancer. *J Clin Oncol* 2013; **31:** 4105–14.
17 Santoro A, Rimassa L, Borbath I, et al. Tivantinib for second-line treatment of advanced hepatocellular carcinoma: a randomised, placebo-controlled phase 2 study. *Lancet Oncol* 2013; **14:** 55–63.
18 Ko JJ, Xie W, Kroeger N, et al. The International Metastatic Renal Cell Carcinoma Database Consortium model as a prognostic tool in patients with metastatic renal cell carcinoma previously treated with first-line targeted therapy: a population-based study. *Lancet Oncol* 2015; **16:** 293–300.
19 Eisen T, Sternberg CN, Robert C, et al. Targeted therapies for renal cell carcinoma: review of adverse event management strategies. *J Natl Cancer Inst* 2012; **104:** 93–113.
20 Motzer RJ, Escudier B, Oudard S, et al. Phase 3 trial of everolimus for metastatic renal cell carcinoma: final results and analysis of prognostic factors. *Cancer* 2010; **116:** 4256–65.
21 McKay RR, Kroeger N, Xie W, et al. Impact of bone and liver metastases on patients with renal cell carcinoma treated with targeted therapy. *Eur Urol* 2014; **65:** 577–84.
22 Choueiri TK, Pal SK, McDermott DF, et al. A phase I study of cabozantinib (XL184) in patients with renal cell cancer. *Ann Oncol* 2014; **25:** 1603–08.
23 Graham TJ, Box G, Tunariu N, et al. Preclinical evaluation of imaging biomarkers for prostate cancer bone metastasis and response to cabozantinib. *J Natl Cancer Inst* 2014; **106:** dju033.
24 Ciamporcero E, Miles KM, Adelaiye R, et al. Combination strategy targeting VEGF and HGF/c-met in human renal cell carcinoma models. *Mol Cancer Ther* 2015; **14:** 101–10.
25 Motzer RJ, Hutson TE, Glen H, et al. Lenvatinib, everolimus, and the combination in patients with metastatic renal cell carcinoma: a randomised, phase 2, open-label, multicentre trial. *Lancet Oncol* 2015; **16:** 1473–82.
26 Motzer RJ, Hutson TE, Tomczak P, et al. Sunitinib versus interferon alfa in metastatic renal-cell carcinoma. *N Engl J Med* 2007; **356:** 115–24.
27 Escudier B, Eisen T, Stadler WM, et al. Sorafenib for treatment of renal cell carcinoma: final efficacy and safety results of the phase III treatment approaches in renal cancer global evaluation trial. *J Clin Oncol* 2009; **27:** 3312–18.
28 Sternberg CN, Davis ID, Mardiak J, et al. Pazopanib in locally advanced or metastatic renal cell carcinoma: results of a randomized phase III trial. *J Clin Oncol* 2010; **28:** 1061–68.
29 Rini BI, Escudier B, Tomczak P, et al. Comparative effectiveness of axitinib versus sorafenib in advanced renal cell carcinoma (AXIS): a randomised phase 3 trial. *Lancet* 2011; **378:** 1931–39.
30 Motzer RJ, Escudier B, Oudard S, et al. Efficacy of everolimus in advanced renal cell carcinoma: a double-blind, randomised, placebo-controlled phase III trial. *Lancet* 2008; **372:** 449–56.

THE LANCET Oncology

Supplementary appendix

This appendix formed part of the original submission and has been peer reviewed. We post it as supplied by the authors.

Supplement to: Choueiri TK, Escudier B, Powles T, et al, for the METEOR investigators. Cabozantinib versus everolimus in advanced renal cell carcinoma (METEOR): final results from a randomised, open-label, phase 3 trial. *Lancet Oncol* 2016; published online June 5. http://dx.doi.org/10.1016/S1470-2045(16)30107-3.

Supplementary Appendix

Supplement to: Choueiri TK, Escudier B, Powles T, et al. Cabozantinib versus everolimus in advanced renal cell carcinoma (METEOR): final results from a randomised, open-label, phase 3 trial.

Table of Contents

List of participating sites 2

Others Involved in METEOR Study Conduct 5

Table S1. Memorial Sloan Kettering Cancer Center (MSKCC) Prognostic Criteria for Previously Treated Patients with Renal Cell Carcinoma[1] 6

Figure S1. Log of Negative Log of Estimated Survivor Functions 6

Table S2: Kaplan-Meier Estimates of Percent of Patients Alive at Selected Landmarks 6

Figure S2: Kaplan-Meier Plots of Overall Survival for Subgroups of Tumour MET Expression 7

Table S3: Subsequent Anticancer Therapies 8

Table S4: Progression-Free Survival as of the May 22, 2015 Cutoff Date 9

Table S5: Tumour Response as of the May 22, 2015 Cutoff Date 9

Table S6: Adverse Events as of the December 31, 2015 Cutoff Date. 10

References 15

List of participating sites
Institute (Country) Investigator [Number of patients]

Institut Gustave Roussy (France) Bernard Escudier [23]
Dana Farber Cancer Institute (United States) Toni Choueiri [22]
HOCA @ Mater (Australia) Paul Mainwaring [16]
University of Texas MD Anderson Cancer Center (United States) Nizar Tannir [15]
Memorial Sloan Kettering Cancer Center (United States) Robert Motzer [13]
Cleveland Clinic (United States) Brian Rini [12]
Aarhus Universitets Hospital (Denmark) Frede Donskov [11]
Johns Hopkins Hospital (United States) Hans Hammers [11]
Helsingin Yliopistollinen Keskussairaala (Finland) Katriina Peltola [10]
Asan Medical Center (Korea, Republic of) JaeLyun Lee [10]
Washington University (United States) Bruce Roth [10]
Texas Oncology, P.A. (United States) Thomas Hutson [10]
Sunnybrook Research Institute - University of Toronto (Canada) Georg Bjarnason [9]
Országos Onkológiai Intézet (Hungary) Lajos Géczi [9]
Seoul National University Hospital (Korea, Republic of) Bhumsuk Keam [9]
Hospital de La Santa Creu i Sant Pau (Spain) Jose Pablo Maroto [9]
St Bartholomews Hospital (United Kingdom) Thomas Powles [9]
UZ Leuven (Belgium) Patrick Schöffski [8]
Fakultni nemocnice Olomouc (Czech Republic) Bohuslav Melichar [8]
British Columbia Cancer Agency (Canada) Christian Kollmannsberger [7]
Copernicus PL Sp. z o.o. Wojewodzkie Centrum Onkologii (Poland) Joanna Pikiel [7]
Westmead Hospital (Australia) Howard Gurney [6]
Fundación Arturo López Pérez (FALP) (Chile) Pamela Salman [6]
Centro Internacional de Estudios Clínicos (Chile) Osvaldo Arén Frontera [6]
Herlev Hospital (Denmark) Poul Geertsen [6]
Hôpital Saint-André (France) Marine Gross-Goupil [6]
Severance Hospital at Yonsei University Health System (Korea, Republic of) Sun Young Rha [6]
Samsung Medical Center (Korea, Republic of) Se Hoon Park [6]
Russian Oncology Research Center n a N N Blokhin (Russian Federation) Dmitry Nosov [6]
Hospital Universitario Ramon y Cajal (Spain) Enrique Grande Pulido [6]
Hospital Universitario Vall d'Hebron (Spain) Cristina Suarez [6]
Ohio State University (United States) Thomas Olencki [6]
Huntsman Cancer Institute at The University of Utah (United States) Neeraj Agarwal [6]
Hôpital Européen Georges Pompidou (France) Stéphane Oudard [5]
Centre René Gauducheau Centre de Lutte Contre Le Cancer Nantes Atlantique (France) Frederic Rolland [5]
Presidio Ospedaliero San Donato (Italy) Sergio Bracarda [5]
Szpital Kliniczny Przemienienia Panskiego Uniwersytetu Medycznego im. K. Marcinkowskiego w Poznaniu (Poland) Piotr Tomczak [5]
Hospital Universitario 12 de Octubre (Spain) Daniel Castellano [5]
University of Pittsburgh Medical Center (United States) Leonard Appleman [5]
Medical University of South Carolina (United States) Harry Drabkin [5]
University of Iowa Hospitals and Clinics (United States) Daniel Vaena [5]
St George Private Hospital (Australia) Paul de Souza [4]
Monash Medical Centre - Moorabbin Campus (Australia) David Pook [4]
Princess Margaret Hospital (Canada) Jennifer Knox [4]
Cross Cancer Institute (Canada) Naveen Basappa [4]
Alberta Health Services (Canada) Daniel Heng [4]
Institut Paoli Calmettes (France) Gwenaëlle Gravis [4]
LMU Klinikum der Universität München (Germany) Michael Staehler [4]
Universitätsklinikum Hamburg Eppendorf (Germany) Michael Rink [4]
Klinikum rechts der Isar der Technischen Universität München (Germany) Margitta Retz [4]
Jasz-Nagykun-Szolnok Megyei Hetenyi Geza Korhaz-Rendelointezet (Hungary) Tibor Csoszi [4]

Mater Misericordiae University Hospital (Ireland) John McCaffrey [4]
Istituto Scientifico Romagnolo Per Lo Studio E La Cura Dei Tumori IRST (Italy) Ugo De Giorgi [4]
Azienda Ospedaliera S Maria Di Terni (Italy) Fausto Roila [4]
Regional Clinical Oncology Hospital (Russian Federation) Sergey Cheporov [4]
Hospital Universitario Virgen del Rocio (Spain) Ignacio Duran [4]
Hospital Universitario Central de Asturias (Spain) Emilio Esteban Gonzalez [4]
Karolinska Universitetssjukhuset Solna (Sweden) Ulrika Harmenberg [4]
Royal Marsden Hospital (United Kingdom) James Larkin [4]
Sarah Cannon Research Institute UK (United Kingdom) Simon Chowdhury [4]
Fox Chase Cancer Center (United States) Daniel Geynisman [4]
Duke University Medical Center (United States) Daniel George [4]
University of Miami (United States) Jaime Merchan [4]
University of Michigan Comprehensive Cancer Center (United States) Bruce Redman [4]
Oregon Health and Science University (United States) Christopher Ryan [4]
Comprehensive Cancer Centers of Nevada (United States) Oscar Goodman [4]
City of Hope National Medical Center (United States) Sumanta Pal [4]
Mayo Clinic Arizona (United States) Thai Ho [4]
University of Arizona (United States) Parminder Singh [4]
Prince of Wales Hospital (Australia) Elizabeth Hovey [3]
Royal Hobart Hospital (Australia) Louise Nott [3]
Princess Alexandra Hospital (Australia) Katharine Cuff [3]
Medizinische Universitat Wien (Austria) Manuela Schmidinger [3]
Institute Jules Bordet (Belgium) Thierry Gil [3]
Nova Scotia Cancer Centre (Canada) Lori Wood [3]
Lakeridge Health Center (Canada) Pawel Zalewski [3]
Fakultni nemocnice u sv. Anny v Brne (Czech Republic) Jana Katolicka [3]
Odense Universitetshospital (Denmark) Niels Viggo Jensen [3]
IUCT-Oncopôle /Institut Claudius Regaud (France) Christine Chevreau [3]
Centre Jean Bernard Clinique Victor Hugo (France) Eric Voog [3]
Centre Léon Bérard Centre Régional de Lutte Contre Le Cancer Rhône Alpes (France) Sylvie Negrier [3]
Onkodok GmbH (Germany) Reinhard Depenbusch [3]
Universitätsklinikum Frankfurt (Germany) Lothar Bergmann [3]
National Center for Tumor Diseases NCT Heidelberg (Germany) Carsten Grüllich [3]
Tallaght Hospital (Ireland) Raymond McDermott [3]
Azienda Ospedaliera San Camillo Forlanini (Italy) Cora Sternberg [3]
Het Nederlands Kanker Instituut Antoni Van Leeuwenhoek Ziekenhuis (Netherlands) Johannes van Thienen [3]
Wojskowy Instytut Medyczny Centralny Szpital Kliniczny MON (Poland) Cezary Szczylik [3]
Hospital da Luz (Portugal) José Passos Coelho [3]
Clinical Oncology Dispensary (Russian Federation) Evgeniy Kopyltsov [3]
Instituto Catalan de Oncologia de Hospitalet (ICO) (Spain) Xavier García del Muro Solans [3]
Hospital Universitario HM Sanchinarro-CIOCC (Spain) Jesús García-Donás [3]
Gazi Universitesi Tip Fakultesi (Turkey) Ramazan Yildiz [3]
University of South Florida (United States) Mayer Fishman [3]
Mayo Clinic (United States) Brian Costello [3]
University of Chicago (United States) Walter Stadler [3]
Northwestern University (United States) Timothy Kuzel [3]
University of Kansas Cancer Center and Medical Pavillion (United States) Stephen Williamson [3]
Royal Adelaide Hospital (Australia) Hsiang Tan [2]
Sydney Adventist Hospital (Australia) Gavin Marx [2]
Box Hill Hospital (Australia) Ian Davis [2]
Port Macquarie Base Hospital (Australia) Stephen Begbie [2]
Kaiser Franz Josef Spital - SMZ Süd (Austria) Maria De Santis [2]
Imelda VZW (Belgium) Wim Wynendaele [2]
Algemeen Ziekenhuis Klina (Belgium) Wim Demey [2]
Juravinski Cancer Centre (Canada) Sebastien Hotte [2]
London Health Sciences Centre (Canada) Eric Winquist [2]

Centre Eugene Marquis Centre Regional de Lutte Contre Le Cancer (France) Brigitte Laguerre [2]
Hopital Jean Minjoz (France) Tristan Maurina [2]
Universitätsklinikum der RWTH Aachen (Germany) Maria Angerer-Shpilenya [2]
Medizinische Hochschule Hannover (Germany) Viktor Grünwald [2]
Universitätsklinikum Freiburg (Germany) Wolfgang Schultze-Seemann [2]
Azienda Socio Sanitaria Territoriale di Cremona (Italy) Rodolfo Passalacqua [2]
Azienda Ospedaliero Universitaria Di Modena Policlinico (Italy) Roberto Sabbatini [2]
Ospedale Di Faenza (Italy) Francesco Carrozza [2]
Bialostockie Centrum Onkologii im. Marii Sklodowskiej-Curie w Bialymstoku (Poland) Marek Wojtukiewicz [2]
Fakultna nemocnica s poliklinikou Zilina (Slovakia) Juraj Mikulas [2]
Clinica Universitaria de Navarra (Spain) José Luis Pérez Gracia [2]
Hospital Regional Universitario de Malaga - Hospital Universitario Virgen de la Victoria (Spain) Maria Isabel Saez Medina [2]
Skanes Universitetssjukhus Lund (Sweden) René Blom [2]
National Taiwan University (Taiwan, Province of China) Chia-Chi Lin [2]
Veterans General Hospital-Taipei (Taiwan, Province of China) Yen-Hwa Chang [2]
Taichung Veterans General Hospital (Taiwan, Province of China) Yen-Chuan Ou [2]
Christie Hospital (United Kingdom) Robert Hawkins [2]
Sarah Cannon Cancer Center (United States) John Hainsworth [2]
University of Alabama at Birmingham (United States) Guru Sonpavde [2]
University of Maryland Greenebaum Cancer Center (United States) Arif Hussain [2]
Advanced Medical Specialties (United States) Michael Troner [2]
Karmanos Cancer Institute (United States) Ulka Vaishampayan [2]
Texas Oncology (United States) Gurjyot Doshi [2]
University of Texas Health Science Center San Antonio (United States) John Sarantopoulos [2]
University of Washington (United States) Scott Tykodi [2]
University of California Los Angeles (United States) Fairooz Kabbinavar [2]
Cedars Sinai Medical Center (United States) Robert Figlin [2]
Border Medical Oncology (Australia) Christopher Steer [1]
St Vincent's Hospital Sydney (Australia) Richard Epstein [1]
Krankenhaus der Barmherzigen Schwestern Linz (Austria) Wolfgang Loidl [1]
CHUM Notre Dame Hospital (Canada) Denis Soulieres [1]
Cancer Care Manitoba (Canada) Piotr Czaykowski [1]
Centre François Baclesse (France) Florence Joly-Lobbedez [1]
Universitätsklinikum Carl Gustav Carus an der TU Dresden (Germany) Manfred Wirth [1]
Universitätsklinikum Ulm (Germany) Thomas J Schnöller [1]
IRCCS Azienda Ospedaliera Universitaria San Martino Istituto Nazionale Per La Ricerca Sul Cancro (Italy) Francesco Boccardo [1]
Università Campus Bio Medico Di Roma (Italy) Daniele Santini [1]
Academisch Ziekenhuis Maastricht (Netherlands) Maureen Aarts [1]
Leids Universitair Medisch Centrum (Netherlands) Susanne Osanto [1]
Instituto Portugues de Oncologia Do Porto Francisco Gentil Epe (Portugal) Nuno Sousa [1]
Centro Hospitalar Lisboa Norte, E.P.E. – Hospital de Santa Maria (Portugal) Luis Costa [1]
Milab sro (Slovakia) Ivan Mincik [1]
Hospital Clinico Universitario de Valencia (Spain) Isabel Chirivella [1]
Hospital del Mar (Spain) Alejandro Martinez [1]
Norrlands Universitetssjukhus (Sweden) Marcus Thomasson [1]
Gaziantep Universitesi Tip Fakultesi Onkoloji Hastanesi (Turkey) Alper Sevinc [1]
Marmara Universitesi Pendik Egitim ve Arastirma Hastanesi (Turkey) Faysal Dane [1]
Ege Universitesi Tip Fakultesi Hastanesi (Turkey) Erhan Gokmen [1]
Clatterbridge Centre for Oncology NHS Foundation Trust Clatterbridge Centre for Oncology - Wirral (United Kingdom) Syed Hussain [1]
Mount Vernon Hospital (United Kingdom) Paul Nathan [1]
Queen Elizabeth Hospital (United Kingdom) Emilio Porfiri [1]
Western General Hospital (United Kingdom) Jahangeer Malik [1]
Aberdeen Royal Infirmary (United Kingdom) Alan MacDonald [1]

Royal Marsden Hospital (United Kingdom) James Larkin [1]
Beatson West of Scotland Cancer Centre (United Kingdom) Hilary Glen [1]
Yale University School of Medicine (United States) Harriet Kluger [1]
New York Oncology Hematology, PC (United States) David Shaffer [1]
Lynn Cancer Institute (United States) Alan Koletsky [1]
University of Tennessee Medical Center (United States) Wahid Hanna [1]
Texas Oncology (United States) Jennifer Wright [1]
Texas Oncology (United States) Stephen Richey [1]
The Permanente Medical Group Inc (United States) Tatjana Kolevska [1]
Banner MD Anderson Cancer Center (United States) Bryan Wong [1]
University of California San Diego - Moores Cancer Center (United States) James Randall [1]

Others Involved in METEOR Study Conduct

Members of the METEOR Steering Committee: Toni K. Choueiri, MD, Bernard Escudier, MD, Thomas Powles, MD, and Robert J. Motzer, MD.

Members of the METEOR Independent Data Monitoring Committee: Ronald Bukowski, MD, Primo N. Lara, Jr., MD, Ronald de Wit, MD, PhD, Daniel Sargent, PhD.

Staff of the sponsor involved in data collection and analyses: Julie Lougheed, Milan Mangeshkar, Maggie Parker, Steve Milwee, Douglas Clary, Heather Niemi, Michelle Van Ryswyk, Tracey Ziomek, Izzy Cornelio, Alfonso Kondo, James Lenihan, Xiaochun Yao, Mark Dean, Jill Youkstetter, Jo-Wei Wan, Alan Arroyo

Table S1. Memorial Sloan Kettering Cancer Center (MSKCC) Prognostic Criteria for Previously Treated Patients with Renal Cell Carcinoma[1]

Number of Risk Factors*	Expected Outcome
0	Favorable
1	Intermediate
2 or 3	Poor

*The three risk factors are:
Karnofsky performance status score < 80%
Hemoglobin < 13 g/dL for males or < 11.5 g/dL for females
Corrected calcium > upper limit of normal

Figure S1. Log of Negative Log of Estimated Survivor Functions

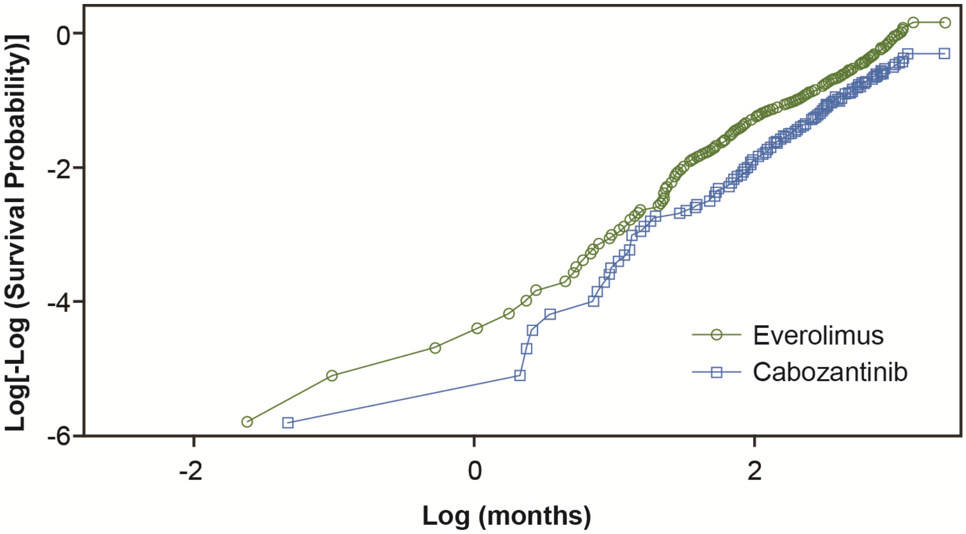

Table S2: Kaplan-Meier Estimates of Percent of Patients Alive at Selected Landmarks

Landmark	Estimate of % of Patients Alive with 95% CI	
	Cabozantinib N=330	Everolimus N=328
6 Months	91% (87%, 93%)	81% (76%, 85%)
12 Months	73% (68%, 79%)	63% (58%, 78%)
18 Months	58% (53%, 64%)	47% (41%, 52%)
24 Months	48% (39%, 55%)	31% (23%, 39%)

All 658 randomised patients were included in the analysis.

Figure S2: Kaplan-Meier Plots of Overall Survival for Subgroups of Tumour MET Expression

High MET Expression

	No. of Patients	Median Overall Survival (95% CI) *months*	No. of Deaths
**Cabozantinib**	51	22·0 (15·4-NE)	20
**Everolimus**	50	15·2 (8·7-NE)	27

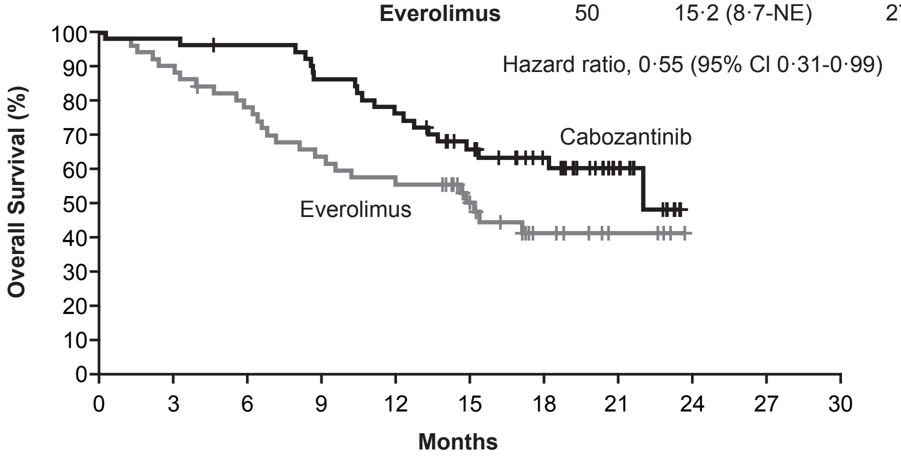

No. at Risk									
Cabozantinib	51	50	48	43	38	29	20	8	0
Everolimus	50	45	38	31	27	18	9	4	0
**No. Censored**									
Cabozantinib	0	0	1	0	0	4	8	11	7
Everolimus	0	0	1	0	0	7	6	5	4

Low MET Expression

	No. of Patients	Median Overall Survival (95% CI) *months*	No. of Deaths
**Cabozantinib**	150	20·8 (18·1-NE)	63
**Everolimus**	162	18·4 (15·9-19.6)	87

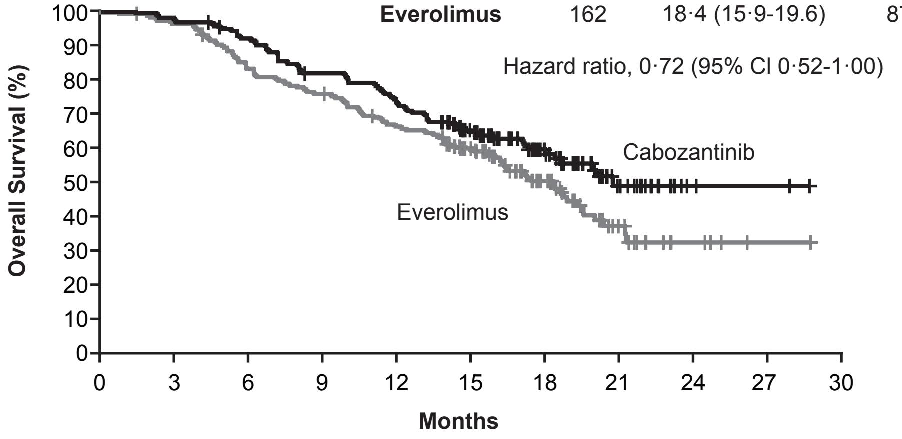

No. at Risk											
Cabozantinib	150	147	136	120	108	80	48	18	3	2	0
Everolimus	162	155	133	121	104	82	48	16	5	1	0
**No. Censored**											
Cabozantinib	0	0	2	1	0	16	26	24	15	1	2
Everolimus	0	1	1	0	2	12	23	22	9	4	1

The number of censored patients is summarized by interval.
CI, confidence interval; NE, not estimable; No, number

Table S3: Subsequent Anticancer Therapies

	Cabozantinib N=330 n (%)	Everolimus N=328 n (%)
Systemic therapy	165 (50)	181 (55)
VEGFR-TKI Therapies	79 (24)	155 (47)
Axitinib	57 (17)	90 (27)
Cabozantinib[a]	0	7 (2)
Pazopanib	5 (2)	22 (7)
Sorafenib	9 (3)	31 (9)
Sunitinib	17 (5)	33 (10)
Other Selected Systemic Therapies		
Everolimus[a]	96 (29)	15 (5)
Temsirolimus	6 (2)	4 (1)
Bevacizumab	8 (2)	11 (3)
Interleukins (Interleukin 2)	0	4 (1)
Interferon-α/Peginterferon	5 (2)	7 (2)
PD-1/PD-L1 targeting agents[b]	15 (5)	19 (6)
Chemotherapy	11 (3)	13 (4)
External beam radiotherapy	61 (18)	77 (23)
Surgery (tumour lesions)	13 (4)	9 (3)

Note: patients may have received more than one type of anticancer therapy.
All 658 randomised patients were included in the analysis.
[a] Refers to commercial use.
[b] 14 patients received nivolumab and one received pembrolizumab. In the everolimus arm, 16 patients received nivolumab, two received AMP-514, and one received atezolizumab.
PD-1, programmed cell death protein-1; PD-L1, programmed cell death ligand-1; TKI, tyrosine kinase inhibitor; VEGFR, vascular endothelial growth factor receptor

Table S4: Progression-Free Survival as of the May 22, 2015 Cutoff Date

	IRC		Investigator	
	Cabozantinib N=330	Everolimus N=328	Cabozantinib N=330	Everolimus N=328
Event, n (%)	180 (55)	214 (66)	196 (59)	233 (71)
Median duration of progression-free survival (95% confidence interval) (months)	7·4 (6·6-9·1)	3·9 (3·7-5·1)	7.4 (7·3-7·8)	5·1 (3·9-5·5)
Stratified p-value	<0·0001		<0·0001	
Hazard ratio (95% confidence interval)	0·52 (0·42-0·64)		0·54 (0·44-0·65)	

All 658 randomised patients were included in the analysis.
Median is based on Kaplan-Meier survival estimates.
Stratification factors were those used for randomisation. The hazard ratio was estimated using the Cox proportional hazard model adjusted for stratification factors. Hazard ratio <1 indicates progression-free survival (PFS) in favour of cabozantinib.
IRC, independent radiology review committee

Table S5: Tumour Response as of the May 22, 2015 Cutoff Date

	Cabozantinib N=330		Everolimus N=328	
	IRC	Investigator	IRC	Investigator
Objective response rate (95% confidence interval)[a]	17 (13-22)[b]	24 (19-29)[b]	3 (2-6)	4 (2-7)
Best overall response, n (%)				
Confirmed complete response	0	0	0	0
Confirmed partial response	57 (17)	78 (24)	11 (3)	14 (4)
Stable disease	216 (65)	209 (63)	203 (62)	205 (63)
Progressive disease	41 (12)	29 (9)	88 (27)	87 (27)
Not evaluable or missing[c]	16 (5)	14 (4)	26 (8)	22 (7)

All 658 patients were included in the analysis.
[a] The proportion of patients achieving an overall response of confirmed complete response or partial response per RECIST version 1.1.
[b] The p-value from Cochran-Mantel-Haenszel test with stratification factors used for randomisation was <0.0001 for both IRC and investigator-determined response compared to everolimus.
[c] No qualifying post-baseline assessment for overall response.
IRC, independent radiology review committee

Table S6: Adverse Events as of the December 31, 2015 Cutoff Date

Event	Cabozantinib (N=331) n (%)			Everolimus (N=322) n (%)		
	Grade 3	Grade 4	Grade 5	Grade 3	Grade 4	Grade 5
Number of subjects with at least one event	210 (63)	25 (8)	26 (8)	167 (52)	26 (8)	25 (8)
Hypertension	49 (15)	0	0	12 (4)	0	0
Diarrhoea	43 (13)	0	0	7 (2)	0	0
Fatigue	36 (11)	0	0	24 (7)	0	0
Palmar-plantar erythrodysaesthesia syndrome	27 (8)	0	0	3 (1)	0	0
Anaemia	19 (6)	0	0	53 (17)	0	0
Asthenia	15 (5)	0	0	8 (2)	0	0
Nausea	15 (5)	0	0	1 (<1)	0	0
Hypokalaemia	14 (4)	2 (1)	0	4 (1)	2 (1)	0
Hyponatraemia	13 (4)	2 (1)	0	7 (2)	1 (<1)	0
Abdominal pain	12 (4)	0	0	5 (2)	0	0
Hypophosphataemia	11 (3)	1 (<1)	0	8 (2)	0	0
Decreased appetite	10 (3)	0	0	3 (1)	0	0
Dyspnoea	10 (3)	0	0	11 (3)	3 (1)	0
Pleural effusion	10 (3)	0	0	7 (2)	0	0
Weight decreased	9 (3)	0	0	0	0	0
Back pain	8 (2)	0	0	7 (2)	0	0
Proteinuria	8 (2)	0	0	2 (1)	0	0
Pulmonary embolism	8 (2)	1 (<1)	0	1 (<1)	0	0
Stomatitis	8 (2)	0	0	7 (2)	0	0
Alanine aminotransferase increased	7 (2)	1 (<1)	0	1 (<1)	0	0
Hypocalcaemia	7 (2)	0	0	0	0	0
Lipase increased	7 (2)	2 (1)	0	3 (1)	0	0
Vomiting	7 (2)	0	0	3 (1)	0	0
Amylase increased	6 (2)	0	0	1 (<1)	1 (<1)	0
Hypomagnesaemia	6 (2)	10 (3)	0	0	0	0
Pneumonia	6 (2)	0	1 (<1)	10 (3)	2 (1)	1 (<1)
Syncope	6 (2)	0	0	2 (1)	0	0
Aspartate aminotransferase increased	5 (2)	0	0	1(<1)	0	0
Blood bilirubin increased	5 (2)	0	0	0	0	0
Hyperkalaemia	5 (2)	0	0	6 (2)	1 (<1)	0
Mucosal inflammation	5 (2)	0	0	10 (3)	1 (<1)	0
Pain in extremity	5 (2)	0	0	1 (<1)	0	0
Bone pain	4 (1)	0	0	1 (<1)	0	0
Gamma-glutamyltransferase increased	4 (1)	0	0	12 (4)	0	0
General physical health deterioration	4 (1)	3 (1)	1 (<1)	7 (2)	0	1 (<1)
Hypertriglyceridaemia	4 (1)	0	0	7 (2)	3 (1)	0
Hypotension	4 (1)	0	0	0	2 (1)	0
Creatinine urine increased	3 (1)	1 (<1)	0	1 (<1)	0	0
Haematuria	3 (1)	0	0	1 (<1)	0	0
Neutropenia	3 (1)	0	0	1 (<1)	0	0
Pain	3 (1)	1 (<1)	0	4 (1)	0	0
Pyrexia	3 (1)	0	0	2 (1)	0	0
Abdominal abscess	2 (1)	0	0	0	0	0
Abdominal pain upper	2 (1)	0	0	0	0	0
Anal abscess	2 (1)	0	0	0	0	0
Bile duct obstruction	2 (1)	0	0	0	0	0
Blood phosphorus decreased	2 (1)	0	0	0	0	0
Blood pressure increased	2 (1)	0	0	0	0	0
Blood triglycerides increased	2 (1)	0	0	4 (1)	1 (<1)	0
Bronchitis	2 (1)	0	0	1 (<1)	0	0
Chest pain	2 (1)	0	0	2 (1)	1 (<1)	0
Confusional state	2 (1)	0	0	1 (<1)	0	0
Convulsion	2 (1)	0	0	0	0	0
Dehydration	2 (1)	0	0	6 (2)	1 (<1)	0
Dysphonia	2 (1)	0	0	0	0	0
Fall	2 (1)	0	0	0	0	0
Hypercalcaemia	2 (1)	1 (<1)	0	3 (1)	3 (1)	0
Hyperglycaemia	2 (1)	1 (<1)	0	16 (5)	0	0
Hypertensive crisis	2 (1)	0	0	0	0	0
Hypoalbuminaemia	2 (1)	0	0	0	0	0

Event	Cabozantinib (N=331) n (%)			Everolimus (N=322) n (%)		
	Grade 3	Grade 4	Grade 5	Grade 3	Grade 4	Grade 5
Malaise	2 (1)	0	0	0	0	0
Metastases to bone	2 (1)	0	0	2 (1)	0	0
Metastases to spine	2 (1)	0	0	3 (1)	0	0
Mobility decreased	2 (1)	0	0	0	0	0
Musculoskeletal chest pain	2 (1)	0	0	4 (1)	0	0
Neutrophil count decreased	2 (1)	0	0	0	0	0
Pancreatitis	2 (1)	0	0	1 (<1)	0	0
Performance status decreased	2 (1)	0	0	0	0	0
Pneumothorax	2 (1)	1 (<1)	0	1 (<1)	0	0
Proctalgia	2 (1)	0	0	0	0	0
Rash	2 (1)	0	0	2 (1)	0	0
Transaminases increased	2 (1)	0	0	0	0	0
Abdominal lymphadenopathy	1 (<1)	0	0	0	0	0
Activated partial thromboplastin time prolonged	1 (<1)	0	0	0	0	0
Acute prerenal failure	1 (<1)	0	0	0	0	0
Acute sinusitis	1 (<1)	0	0	0	0	0
Adrenal insufficiency	1 (<1)	0	0	0	0	0
Anal fistula	1 (<1)	0	0	0	0	0
Anorectal cellulitis	1 (<1)	0	0	0	0	0
Anxiety	1 (<1)	0	0	0	0	0
Aortic dissection	1 (<1)	0	0	0	0	0
Aphasia	1 (<1)	0	0	0	0	0
Appendicitis	1 (<1)	0	0	0	0	0
Arthralgia	1 (<1)	0	0	4 (1)	0	0
Ascites	1 (<1)	0	0	1 (<1)	0	0
Atrial fibrillation	1 (<1)	0	0	2 (1)	0	0
Bacteraemia	1 (<1)	0	0	0	0	0
Bacterial sepsis	1 (<1)	0	0	0	0	0
Blood alkaline phosphatase increased	1 (<1)	0	0	1 (<1)	0	0
Blood bicarbonate increased	1 (<1)	0	0	0	0	0
Blood creatinine increased	1 (<1)	0	0	0	0	0
Blood lactate dehydrogenase increased	1 (<1)	0	0	0	0	0
Blood potassium increased	1 (<1)	0	0	0	0	0
Blood sodium decreased	1 (<1)	0	0	0	0	0
Blood thyroid stimulating hormone increased	1 (<1)	0	0	0	0	0
Carotid artery occlusion	1 (<1)	0	0	0	0	0
Carotid artery thrombosis	1 (<1)	0	0	0	0	0
Cerebral haematoma	1 (<1)	0	0	0	0	0
Cerebrovascular disorder	1 (<1)	0	0	0	0	0
Cholangitis	1 (<1)	0	0	1 (<1)	0	0
Cholangitis acute	1 (<1)	0	0	0	0	0
Cholecystitis acute	1 (<1)	0	0	1 (<1)	0	0
Cognitive disorder	1 (<1)	0	0	0	0	0
Constipation	1 (<1)	0	0	1 (<1)	0	0
Cough	1 (<1)	0	0	3 (1)	0	0
Deep vein thrombosis	1 (<1)	0	0	0	0	0
Dental caries	1 (<1)	0	0	0	0	0
Device occlusion	1 (<1)	0	0	0	0	0
Device related infection	1 (<1)	0	0	0	0	0
Diabetes mellitus inadequate control	1 (<1)	0	0	1 (<1)	0	0
Disseminated intravascular coagulation	1 (<1)	0	0	0	0	0
Disturbance in attention	1 (<1)	0	0	0	0	0
Dizziness	1 (<1)	0	0	0	0	0
Dyspepsia	1 (<1)	0	0	0	0	0
Enterovirus infection	1 (<1)	0	0	0	0	0
Epigastric discomfort	1 (<1)	0	0	0	0	0
Epilepsy	1 (<1)	0	0	0	0	0
Foreign body in eye	1 (<1)	0	0	0	0	0
Fracture	1 (<1)	0	0	0	0	0
Fungal skin infection	1 (<1)	0	0	0	0	0
Gastric haemorrhage	1 (<1)	0	0	1 (<1)	0	0
Gastric ulcer	1 (<1)	0	0	0	0	0

Event	Cabozantinib (N=331) n (%)			Everolimus (N=322) n (%)		
	Grade 3	Grade 4	Grade 5	Grade 3	Grade 4	Grade 5
Gastrointestinal haemorrhage	1 (<1)	0	0	0	0	0
Gastrointestinal perforation	1 (<1)	0	0	0	0	(<1)
Haemoglobin decreased	1 (<1)	0	0	3 (1)	0	0
Headache	1 (<1)	0	0	1 (<1)	0	0
Helicobacter infection	1 (<1)	0	0	0	0	0
Humerus fracture	1 (<1)	0	0	1 (<1)	0	0
Hypochloraemia	1 (<1)	0	0	0	0	0
Hypoglycaemia	1 (<1)	0	0	0	0	0
Hypogonadism	1 (<1)	0	0	0	0	0
Ileus	1 (<1)	0	0	0	0	0
Impaired healing	1 (<1)	0	0	0	0	0
Implant site infection	1 (<1)	0	0	0	0	0
Infection	1 (<1)	0	0	0	0	0
Intracranial venous sinus thrombosis	1 (<1)	0	0	0	0	0
Jaw fracture	1 (<1)	0	0	0	0	0
Kidney infection	1 (<1)	0	0	0	0	0
Klebsiella infection	1 (<1)	0	0	0	0	0
Lethargy	1 (<1)	0	0	0	0	0
Liver function test abnormal	1 (<1)	0	0	0	0	0
Local swelling	1 (<1)	0	0	0	0	0
Loss of consciousness	1 (<1)	0	0	0	0	0
Lower respiratory tract infection	1 (<1)	0	0	2 (1)	0	0
Lung disorder	1 (<1)	0	0	0	0	0
Lung infection	1 (<1)	0	0	1 (<1)	0	0
Lymphadenopathy	1 (<1)	0	0	0	0	0
Lymphocyte count decreased	1 (<1)	0	0	4 (1)	0	0
Lymphopenia	1 (<1)	0	0	6 (2)	0	0
Memory impairment	1 (<1)	0	0	0	0	0
Metabolic acidosis	1 (<1)	0	0	0	0	0
Metastases to central nervous system	1 (<1)	0	0	1 (<1)	1 (<1)	0
Metastases to ovary	1 (<1)	0	0	0	0	0
Metastases to pelvis	1 (<1)	0	0	0	0	0
Metastases to testicle	1 (<1)	0	0	0	0	0
Motor dysfunction	1 (<1)	0	0	0	0	0
Musculoskeletal pain	1 (<1)	0	0	0	0	0
Neck pain	1 (<1)	0	0	0	0	0
Oesophageal pain	1 (<1)	0	0	0	0	0
Oesophagitis	1 (<1)	0	0	0	0	0
Osteolysis	1 (<1)	0	0	0	0	0
Osteomyelitis	1 (<1)	0	0	0	0	0
Osteonecrosis of jaw	1 (<1)	0	0	2 (1)	0	0
Pain of skin	1 (<1)	0	0	0	0	0
Pancreatitis acute	1 (<1)	0	0	0	0	0
Pathological fracture	1 (<1)	0	0	1 (<1)	1 (<1)	0
Pelvic fracture	1 (<1)	0	0	0	0	0
Pelvic pain	1 (<1)	0	0	1 (<1)	0	0
Peripheral ischaemia	1 (<1)	0	0	0	0	0
Platelet count decreased	1 (<1)	0	0	0	1 (<1)	0
Pleuritic pain	1 (<1)	0	0	0	0	0
Portal vein thrombosis	1 (<1)	0	0	1 (<1)	0	0
Presyncope	1 (<1)	0	0	0	0	0
Proctitis	1 (<1)	0	0	0	0	0
Pulmonary thrombosis	1 (<1)	0	0	0	0	0
Renal failure	1 (<1)	0	0	1 (<1)	0	0
Renal pain	1 (<1)	0	0	0	0	0
Respiratory distress	1 (<1)	0	0	0	0	0
Respiratory failure	1 (<1)	0	0	0	0	1 (<1)
Rib fracture	1 (<1)	0	0	0	0	0
Sciatica	1 (<1)	0	0	1 (<1)	0	0
Sepsis	1 (<1)	0	0	1 (<1)	2 (1)	0
Sinusitis	1 (<1)	0	0	0	0	0
Skin ulcer	1 (<1)	0	0	0	0	0
Small intestinal obstruction	1 (<1)	0	0	0	0	0

Event	Cabozantinib (N=331) n (%)			Everolimus (N=322) n (%)		
	Grade 3	Grade 4	Grade 5	Grade 3	Grade 4	Grade 5
Small intestinal perforation	1 (<1)	0	0	0	0	0
Spinal cord compression	1 (<1)	0	0	4 (1)	1 (<1)	0
Spinal fracture	1 (<1)	0	0	1 (<1)	0	0
Splenic vein thrombosis	1 (<1)	0	0	0	0	0
Squamous cell carcinoma	1 (<1)	0	0	0	0	0
Thoracic vertebral fracture	1 (<1)	0	0	0	0	0
Thrombocytopenia	1 (<1)	0	0	2 (1)	0	0
Tooth abscess	1 (<1)	0	0	1 (<1)	0	0
Tumour pain	1 (<1)	0	0	0	0	0
Ulcer haemorrhage	1 (<1)	0	0	0	0	0
Upper respiratory tract infection	1 (<1)	0	0	0	0	0
Urinary tract infection	1 (<1)	0	0	3 (1)	0	0
Urinary tract obstruction	1 (<1)	0	0	0	0	0
Urinary tract pain	1 (<1)	0	0	0	0	0
Urine protein/creatinine ratio increased	1 (<1)	1 (<1)	0	1 (<1)	0	0
Urosepsis	1 (<1)	0	1 (<1)	0	0	0
Vaginal inflammation	1 (<1)	0	0	0	0	0
Vulval cellulitis	1 (<1)	0	0	0	0	0
White blood cell count decreased	1 (<1)	0	0	1 (<1)	0	0
Uncoded: bone infection	1 (<1)	0	0	0	0	0
Uncoded: c2 metastases new lesion	1 (<1)	0	0	0	0	0
Uncoded: cardio arteries blockage	1 (<1)	0	0	0	0	0
Uncoded: fracture -pathologic (medical margin l acetabulum)	1 (<1)	0	0	0	0	0
Uncoded: infection of jaw	1 (<1)	0	0	0	0	0
Uncoded: limp urination	1 (<1)	0	0	0	0	0
Uncoded: osteonecrosis of jaw (left posterior maxilla)	1 (<1)	0	0	0	0	0
Abdominal distension	0	0	0	1 (<1)	0	0
Abdominal infection	0	0	0	1 (<1)	0	0
Abdominal pain lower	0	0	0	1 (<1)	0	0
Abscess neck	0	0	0	1 (<1)	0	0
Acetabulum fracture	0	0	0	1 (<1)	0	0
Acute coronary syndrome	0	0	0	1 (<1)	0	0
Acute respiratory failure	0	0	0	1 (<1)	0	0
Agitation	0	0	0	1 (<1)	0	0
Angioedema	0	0	0	1 (<1)	0	0
Arrhythmia	0	0	0	1 (<1)	0	0
Aspergillus infection	0	0	0	0	0	1 (<1)
Blood alkaline phosphatase	0	0	0	1 (<1)	0	0
Blood calcium increased	0	0	0	0	1 (<1)	0
Blood cholesterol increased	0	1 (<1)	0	1 (<1)	1 (<1)	0
Blood creatine phosphokinase increased	0	1 (<1)	0	0	0	0
Blood glucose increased	0	0	0	1 (<1)	0	0
Blood potassium decreased	0	0	0	1 (<1)	0	0
Bradycardia	0	0	0	1 (<1)	0	0
Bronchopneumonia	0	0	0	1 (<1)	0	0
Cardiac failure	0	0	1 (<1)	0	0	0
Cardiac failure congestive	0	0	0	0	0	1 (<1)
Cardiac tamponade	0	1 (<1)	0	0	1 (<1)	0
Cardiopulmonary failure	0	0	1 (<1)	0	0	0
Cholecystitis	0	0	0	1 (<1)	0	0
Cholecystitis infective	0	0	0	1 (<1)	0	0
Circulatory collapse	0	0	0	0	0	1 (<1)
Colitis	0	0	0	1 (<1)	0	0
Creatine urine increased	0	0	0	1 (<1)	0	0
Death	0	0	2 (1)	0	0	0
Delirium	0	1 (<1)	0	1 (<1)	0	0
Depression	0	0	0	2 (1)	0	0
Diabetes mellitus	0	0	0	3 (1)	0	0
Disease progression	0	0	1 (<1)	0	0	0
Diverticulitis	0	0	0	1 (<1)	0	0
Dysuria	0	0	0	1 (<1)	0	0

Event	Cabozantinib (N=331) n (%)			Everolimus (N=322) n (%)		
	Grade 3	Grade 4	Grade 5	Grade 3	Grade 4	Grade 5
Ear pain	0	0	0	1 (<1)	0	0
Electrocardiogram QT prolonged	0	0	0	1 (<1)	0	0
Electrolyte imbalance	0	1 (<1)	0	0	0	0
Extradural haematoma	0	0	1 (<1)	0	0	0
Eye pain	0	0	0	1 (<1)	0	0
Face oedema	0	0	0	1 (<1)	0	0
Femoral neck fracture	0	0	0	2 (1)	0	0
Flank pain	0	0	0	1 (<1)	0	0
Fungal infection	0	0	0	1 (<1)	0	0
Gastritis	0	0	0	1 (<1)	0	0
Gastroenteritis	0	0	0	1 (<1)	0	0
Generalised oedema	0	0	0	1 (<1)	0	0
Haemarthrosis	0	1 (<1)	0	0	0	0
Haemoptysis	0	0	0	1 (<1)	1 (<1)	0
Haemorrhagic anaemia	0	1 (<1)	0	0	0	0
Haemorrhagic stroke	0	0	0	1 (<1)	0	0
Haemorrhoids	0	0	0	1 (<1)	0	0
Hepatitis cholestatic	0	1 (<1)	0	0	0	0
Herpes zoster	0	0	0	1 (<1)	0	0
Hydrothorax	0	0	0	1 (<1)	0	2 (1)
Hypercalcaemia of malignancy	0	0	0	1 (<1)	0	0
Hypercholesterolaemia	0	1 (<1)	0	0	1 (<1)	0
Hyperlipidaemia	0	0	0	0	1 (<1)	0
Hypersensitivity	0	0	0	1 (<1)	0	0
Hyperuricaemia	0	0	0	1 (<1)	0	0
Hypothyroidism	0	0	0	1 (<1)	0	0
Insomnia	0	0	0	1 (<1)	0	0
Insulin-requiring type 2 diabetes mellitus	0	0	0	1 (<1)	0	0
Interstitial lung disease	0	0	0	2 (1)	0	0
Intestinal obstruction	0	0	0	1 (<1)	0	0
Intestinal perforation	0	0	0	0	1 (<1)	0
Large intestine polyp	0	0	0	1 (<1)	0	0
Left ventricular dysfunction	0	0	0	1 (<1)	0	0
Lumbar spinal stenosis	0	0	0	1 (<1)	0	0
Malignant melanoma	0	0	0	2 (1)	0	0
Malignant neoplasm of orbit	0	0	0	1 (<1)	0	0
Malignant pleural effusion	0	0	0	1 (<1)	0	0
Malnutrition	0	1 (<1)	0	0	0	0
Metastases to peritoneum	0	0	0	0	1 (<1)	0
Metastatic pain	0	0	0	1 (<1)	0	0
Metastatic renal cell carcinoma	0	0	2 (1)	0	0	1 (<1)
Multi-organ failure	0	0	0	0	0	1 (<1)
Muscular weakness	0	0	0	1 (<1)	0	0
Myalgia	0	0	0	1 (<1)	0	0
Myocardial infarction	0	0	0	1 (<1)	0	0
Non-cardiac chest pain	0	0	0	1 (<1)	0	0
Obstructive airways disorder	0	0	0	1 (<1)	0	0
Oedema	0	0	0	1 (<1)	0	0
Oedema peripheral	0	0	0	6 (2)	0	0
Oral disorder	0	0	0	1 (<1)	0	0
Oropharyngeal pain	0	0	0	1 (<1)	0	0
Osteosclerosis	0	0	0	1 (<1)	0	0
Otitis externa	0	0	0	1 (<1)	0	0
Pericardial effusion	0	1 (<1)	0	2 (1)	0	0
Periorbital cellulitis	0	0	0	1 (<1)	0	0
Peripheral motor neuropathy	0	0	0	1 (<1)	0	0
Peritonitis	0	0	0	0	1 (<1)	0
Pleural infection	0	0	1 (<1)	0	0	0
Pneumocystis jirovecii pneumonia	0	0	0	1 (<1)	0	0
Pneumonia aspiration	0	0	0	0	0	3 (1)
Pneumonitis	0	0	0	5 (2)	1 (<1)	0
Post procedural haemorrhage	0	0	1 (<1)	0	0	0
Prostatitis	0	0	0	1 (<1)	0	0

Event	Cabozantinib (N=331) n (%)			Everolimus (N=322) n (%)		
	Grade 3	Grade 4	Grade 5	Grade 3	Grade 4	Grade 5
Pruritus	0	0	0	1 (<1)	0	0
Pyelonephritis	0	0	0	1 (<1)	0	0
Rash generalised	0	0	0	2 (1)	0	0
Rash maculo-papular	0	0	0	1 (<1)	0	0
Renal cancer	0	0	1 (<1)	0	0	1 (<1)
Renal cancer metastatic	0	0	1 (<1)	0	0	0
Renal cell carcinoma	0	0	11 (3)	0	1 (<1)	10 (3)
Renal failure acute	0	0	0	2 (1)	0	0
Renal haemorrhage	0	0	0	0	1 (<1)	0
Renal impairment	0	0	0	1 (<1)	0	0
Respiratory tract infection	0	0	0	1 (<1)	0	0
Septic shock	0	1 (<1)	0	0	0	0
Skin infection	0	0	0	1 (<1)	0	0
Somnolence	0	0	0	2 (1)	0	0
Spinal pain	0	0	0	1 (<1)	0	0
Stress fracture	0	0	0	1 (<1)	0	0
Thrombocytosis	0	0	0	1 (<1)	0	0
Tooth infection	0	0	0	1 (<1)	0	0
Toothache	0	0	0	1 (<1)	0	0
Tuberculosis	0	0	0	1 (<1)	0	0
Tumour thrombosis	0	0	0	1 (<1)	0	0
Type 2 diabetes mellitus	0	0	0	0	1 (<1)	0
Upper gastrointestinal haemorrhage	0	0	0	1 (<1)	0	0
Weight increased	0	0	0	1 (<1)	0	0
Wound infection	0	0	0	1 (<1)	0	0

All grade 3, 4 and 5 adverse events are summarized for both treatment arms. Terms labeled uncoded are verbatim terms that were not coded to preferred terms at the time of the analysis.

References

1. Motzer RJ, Bacik J, Schwartz LH, et al. Prognostic factors for survival in previously treated patients with metastatic renal cell carcinoma. *J Clin Oncol* 2004; **22**: 454-63.
